# MOdified DIagnostic strateGy to safely ruLe-out pulmonary embolism In the emergency depArtment: study protocol for the Non-Inferiority MODIGLIANI cluster cross-over randomized trial

**DOI:** 10.1186/s13063-020-04379-y

**Published:** 2020-06-03

**Authors:** Anne-Laure Philippon, Margaux Dumont, Sonia Jimenez, Sarah Salhi, Marine Cachanado, Isabelle Durand-Zaleski, Tabassome Simon, Yonathan Freund

**Affiliations:** 1 Emergency department, Hôpital Pitié-Salpêtrière, Assistance Publique – Hôpitaux de Paris, APHP, Sorbonne Université, Paris, France; 2 Emergency Department, Hospital Clinic, Barcelona, Spain; 3 Department of clinical pharmacology and Clinical Research Platform of East of Paris (URCEST-CRC-CRB), APHP.Sorbonne Universite, hôpital Saint Antoine, Paris, France; 4 Sorbonne Université, Paris, France; 5 Health economics research unit, Hopital de l’Hotel Dieu APHP, Paris, France

**Keywords:** Emergency department, Pulmonary embolism, D-dimers

## Abstract

**Introduction:**

In the work-up strategy for pulmonary embolism (PE) in the ED, the recently introduced YEARS rule allows the raising of the D-dimer threshold to 1000 ng/ml in patients with no signs of deep venous thrombosis and no hemoptysis and in whom PE is not the most likely diagnosis. However, this decision rule has never been prospectively compared to the usual strategy. Furthermore, it is unclear if the YEARS rule can be used on top of the Pulmonary Embolism Rule-out Criteria (PERC). We aim to assess the non-inferiority of YEARS compared to current guidelines to rule out PE among PERC-positive ED patients with suspicion of PE.

**Methods/design:**

The MODIGLIANI study is a multicenter, European, non-inferiority, cluster-randomized, two periods cross-over, controlled trial. Each center will be randomized for the sequence of two 4-month periods: intervention (MOdified Diagnostic Strategy: MODS) followed by control (usual care), or control followed by intervention with 1 month of “wash-out” between the two periods. In the control period, the threshold will be as usual (500 ng/ml for patients aged 50 years or younger and age × 10 for older patients). In the MODS period, the threshold of D-dimers to rule out PE will be raised to 1000 ng/ml if no item of the YEARS score is present or will remain unchanged otherwise. Patients will be included if they have a suspicion of PE, defined as chest pain, dyspnea, or syncope. Non-inclusion criteria comprise a high clinical probability of PE or PERC-negative patients with low clinical probability.

**Ethics and dissemination:**

The study has received the following approvals: Comité de protection des personnes Ile de France XI (France) and Comité de Ética de la Investigación con medicamentos del Hospital Clínic de Barcelona (Spain). Results will be made available to all included participants and other researchers.

**Trial registration:**

ClinicalTrials.gov, NCT04032769. Registered on 24 July 2019.

## Strengths and limitation of this study

- This trial will address the safety of the YEARS rule applied in combination with PERC (i.e., after excluding PERC-negative patients) for the exclusion of pulmonary embolism in the emergency department.
- This trial will compare and assess the benefits of YEARS compared to the conventional strategy with age-adjusted D-dimers.
- This trial is a cluster cross-over randomized trial and is not randomized at the patient level.

## Background

The diagnosis of pulmonary embolism (PE) is a crucial matter in the emergency department (ED) [1]. The overall prevalence of PE in suspected patients continues to decrease, and the rate of diagnostic failure is now below 1% in Europe and the USA [2, 3].. Because a missed PE could be potentially lethal, several researches reported that PE is both over-investigated and over-diagnosed. The diagnostic gold standards for PE are the computed tomographic pulmonary angiogram (CTPA) and the V/Q scan, which have been shown to have clear risks (allergic reaction, acute renal failure, delayed solid tumor) and other downsides such as prolonged ED stay and increased cost [4–6]. To limit the use of imaging studies, two rules for excluding PE were recently reported to be safe: the PERC rule and the YEARS rule. PERC is an eight-item block of clinical criteria that has recently been validated to safely exclude PE in low-risk patients (Table 1). YEARS is a clinical rule that allows, in the absence of YEARS criteria, to safely raise the threshold of D-dimers to 1000 ng/ml for imaging studies (CTPA or V/Q scan) [7]. However, the latter has only been reported in prospective ED cohorts but has not yet been evaluated compared to the standard practice that follows international guidelines with the usual thresholds for D-dimers (e.g., 500 ng/ml for patients aged 50 years or younger and age × 10 ng/ml for older patients) [7–9]. Therefore, its added value in clinical practice remains uncertain. Furthermore, whether a modified diagnostic algorithm that combines these two rules could safely reduce imaging study use in the ED is unknown.

**Table 1:** Pulmonary Embolism Rule out Criteria (PERC), 0–8, one point for each positive item

Age > 50 years	+ 1 point
Heart rate > 100	+ 1 point
SaO2 < 95%	+ 1 point
Unilateral leg swelling	+ 1 point
Hemoptysis	+ 1 point
Recent trauma or surgery	+ 1 point
History of PE or DVT	+ 1 point
Exogenestrogen use	+ 1 point

*DVT* deep venous thrombosis, *PE* pulmonary embolism

The MODIGLIANI study is a trial designed to assess the non-inferiority and cost-effectiveness of the modified diagnostic strategy (MODS) that combines YEARS and PERC for ruling out PE in the ED.

## Methods/design

### Study design

The MODIGLIANI study is a prospective, multicenter, non-inferiority, cluster-randomized, cross-over, controlled trial in 20 EDs in France and Spain. The complete protocol can be seen in the appendix (Supplementary file 1). The study start date was October 1st 2019 and the end of the recruitment period is estimated to be in June 2020 and study completion October 1st 2020.

**Fig. 1:**
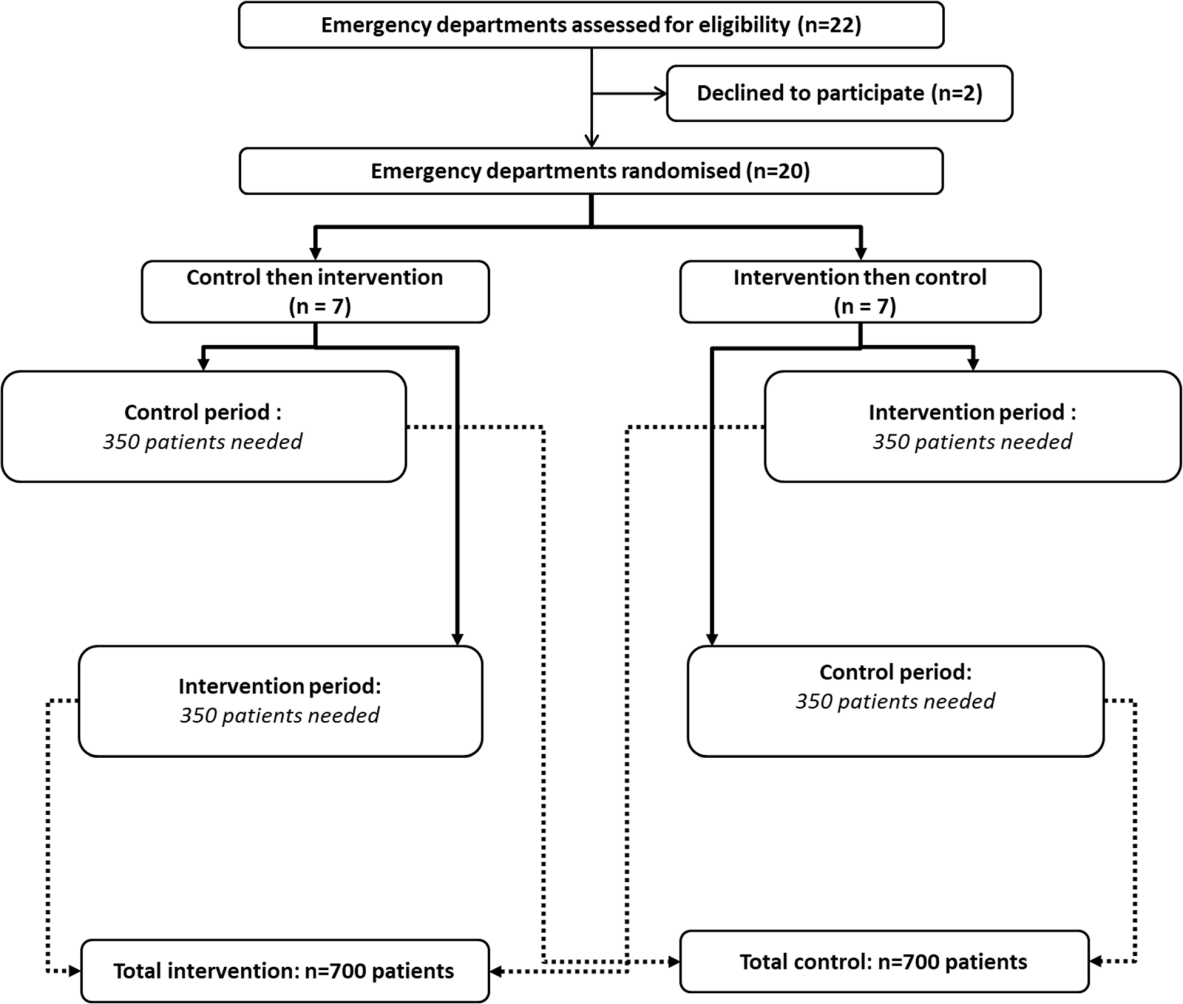
Study flow diagram. Number of subjects needed in each period and strategy

**Fig. 2:**
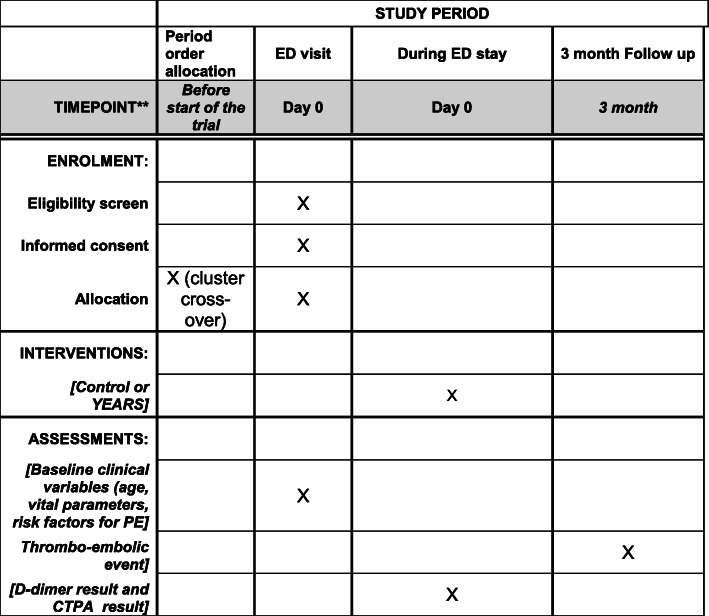
SPIRIT figure. *ED* emergency department, *PE* pulmonary embolism, *CTPA* computed tomographic pulmonary angiogram

Each center will be randomized for the sequence of the periods: intervention (MOdified Diagnostic Strategy (MODS)) followed by control (usual care), or control followed by intervention with 1 month of “wash-out” between the two periods. The participating centers will implement the first assigned strategy until a target number of consecutive patients is reached (half of the patients to be included in total, namely 350 patients in each strategy); in the cross-over phase, the centers will implement the second assigned strategy in a similar number of consecutive patients. The reporting of this study will follow the CONSORT statement extended to cluster randomized trials. The reporting of the trial protocol paper follows the SPIRIT recommendations (Figs. 1 and 2) [10, 11].

### Selection of participants

This study will include patients who provide written or oral consent with a suspicion of PE defined as:

- New onset of or worsening shortness of breath
- or chest pain,
- or syncope

As the prevalence of PE in patients with isolated syncope is not reportedly sufficiently low to rule out PE, we also included this inclusion criteria along with chest pain and shortness of breath [12].

Non-inclusion criteria are listed as follows:

- Anticipated inability to be followed up at 3 months
- Obvious cause other than PE for chest pain, syncope, or dyspnea
- High clinical probability of PE (estimated by the physician gestalt as > 50%)
- Low clinical probability of PE (estimated by the physician gestalt as < 15%) and no item of the PERC score (Table 1)
- Acute severe presentation (clinical signs of respiratory distress, hypotension, SpO2 < 90%, shock)
- Concurrent anticoagulation treatment
- Current diagnosed thrombo-embolic event (in the past 6 months)
- Prisoners
- Pregnancy
- No social security
- Participation in another intervention trial

The emergency physician will assess the clinical probability of PE during screening, estimated by the clinician gestalt. As described in previous studies, this gestalt assessment is established by answering the question “How do you estimate the pre-test clinical probability: low, moderate, or high?” Patients with a high clinical probability of PE will not be included because they should be investigated with CTPA or V/Q scan, and therefore our modified algorithm will not concern them [13–15]. We will also exclude patients with a low clinical probability and a PERC score of 0 (based on clinical parameters taken by the emergency physician at the time of first clinical evaluation), as it has been validated that these patients have a very low risk of PE and should not be investigated for PE [16, 17].

In all participating centers, patients will be screened and recruited by the treating emergency physicians.

### Procedure and interventions

The diagnostic strategy for PE will first include a D-dimer analysis. The threshold for ruling out PE before ordering an imaging study will depend on the study period; in the control period, a positive D-dimer is defined as > 500 ng/ml in patients aged 50 years and younger, and as > age × 10 ng/ml for patients older than 50 years.

In the intervention period, the YEARS score will be assessed (Table 2). If the YEARS score is 0 (absence of any of the three items), a positive D-dimer is defined as > 1000 ng/ml. In other cases, the threshold will remain unchanged (> 500 ng/ml in patients aged 50 years and younger, and as > age × 10 ng/ml for patients older than 50 years).

**Table 2:** YEARS rule, 0–3, one point for each positive item

Hemoptysis	+ 1 point
Clinical signs of DVT	+ 1 point
PE is the most likely diagnosis	+ 1 point

*DVT* deep venous thrombosis, *PE* pulmonary embolism

The total duration of a patient’s participation in the study is 3 months.

### Objectives and outcomes

The primary objective of this trial is to assess the safety of a modified diagnostic strategy (MODS) in the ED using the YEARS rule for patients in whom PE is not excluded by PERC score. Secondary objectives are to assess the efficacy of the MODS strategy in reducing ordering of CTPA or V/Q scan, introduction of anticoagulant, ED length of stay, hospital admission following ED visit, all causes readmission and mortality at 3 months, and diagnosed pulmonary embolism at 3 months after excluding isolated sub-segmental PE. The trial will also assess the safety of the PEPS score (Table 3) and evaluate the overall 3-month cost-reduction of the MODS strategy [18].

**Table 3:** Pulmonary Embolism Probability Score (PEPS)

	Points
Age < 50 years	−2
Age 50–64 years	−1
Chronic respiratory disease	−1
Heart rate < 80/min	− 1
Chest pain and dyspnea	1
Prolonged decubitus	2
Sex male	2
Syncope	2
History of thrombo-embolic disease	2
Estrogen use	2
SpO2 < 95%	3
Lower leg pain or edema	3
Pulmonary embolism is the most likely diagnosis	5

< 0 Pulmonary embolism (*PE*) unlikely (PE ruled out) ; 0–4, low probability (D-dimer threshold at 1000 ng/ml) ; 5–11, intermediate probability (age-adjusted threshold for D-dimer) ; > 11, high probability (computed tomographic pulmonary angiogram indicated)

The primary endpoint is the failure proportion of the diagnostic strategy, defined as a diagnosed thrombo-embolic event at 3 months follow-up (either a PE or a deep venous thrombosis), among patients in whom PE was initially ruled out. Exclusion of PE in the ED is done upon a negative D-dimer result or negative CTPA or negative V/Q scan in both groups. The primary criterion of thrombo-embolic event will be based on an objective diagnosis of DVT on Doppler ultrasonography, an intraluminal defect on CTPA, or a V/Q lung scan with a reported high probability. To confirm the occurrence of the primary endpoint, all files with evidence of thrombo-embolic event collected by the local investigator of each center will be independently reviewed by an adjudication committee of three experts, emergency physicians, blinded one to each other, and blinded to the study period. The adjudication committee will also review cases of death with no evidence of thrombo-embolic event and will adjudicate the death as likely related to a PE or not. Adjudication of a PE-related death will follow the recommendations of Tritschler et al. [19]. The review process includes analysis of all medical files of the patient from inclusion to follow up, including reports of any imaging study.

Secondary endpoints include:

- CTPA or V/Q scan
- Anticoagulant therapy administration
- Length of stay in the ED (hours)
- Admission to the hospital following ED visit
- All causes re-hospitalization at 3 months
- Death from all causes at 3 months
- Diagnosed pulmonary embolism at 3-month follow-up, excluding the isolated sub-segmental pulmonary embolism, among patients in whom PE has been initially ruled out
- Pulmonary Embolism Probability Score (PEPS)
- 3-month total cost and cost-effectiveness (cost per major adverse event averted)

Details of PEPS are reported in Table 3 [18].

### Sample size calculation

In the recent recommendations of the International Society of Thrombosis and Hemostasis on studies for PE diagnosis in the ED, it has been suggested that the maximum acceptable failure rate of a tested strategy should not exceed 1.85% [3]. The recent large European prospective cohort studies on PE diagnosis (PROPER, PERCEPIC, YEARS, ADJUST-PE) reported a failure rate of 0.1–0.5%. With an anticipated failure rate of 0.5% in the control group, a non-inferiority margin set at 1.35% (according to the ISTH recommendations so the upper bound of the 95% confidence interval (CI) of the failure rate in the intervention group will not exceed 1.85%), beta = 20%, and one-sided alpha = 2.5%, N1 = 857 patients are needed (East 6, Cytel, Cambridge, MA, USA). Under the assumption of an intra-class correlation coefficient (CCIC) of 0.018, an interperiod correlation (η) of 0.0115 (based on previous cluster randomized trials in French EDs) and a mean cluster size for one period (m) = 22 patients, the cluster design effect would be 1.37 [17, 20]. Considering 5% of non-evaluable patients, with 18 centers and two periods, 1234 patients are needed.

Besides the non-inferiority analysis, it is of utmost importance that the upper bound of the 95% CI of the failure rate of the tested modified diagnostic strategy remains below 1.85%, whatever the rate of the control group is. Our retrospective study reported an anticipated failure rate at 0.85% but focused only on low risk patients. Therefore, with an anticipated failure rate of 1% in the intervention period, the sample size of this group should be at least 700 to respect the maximal upper bound of 1.85%.

Therefore, for a conservative approach to these two conditions (non-inferiority margin at 1.35% and maximal upper bound of the 95% CI below 1.85%), 1400 (700 in each group) subjects will be needed in this study.

### Randomization and period allocation

Sites’ sequence randomization will be computer generated by a biostatistician from the clinical research platform URC-Est, independent of the study and before the study starts. Randomization will be stratified on country and center size.

### Data collection and data management

Data will be collected in an electronic case report form (e-CRF by the ED investigators with the help of clinical research technicians (CRT)). This e-CRF comprises baseline characteristics (past medical history, systolic blood pressure, heart rate, and other items from PERC, Wells and YEARS decision rules). Follow-up at 3 months will be done by phone interview of the patient or their general practitioner, outpatient consultation, email, or hospital visit. Outcome data recorded at follow-up will be entered in the same e-CRF as any serious adverse events that might occur.

### Statistical analysis plan

The complete statistical plan can be seen in the appendix (Supplementary file 2).

No interim analysis is planned. Analysis will be performed at the end of the study after data review and freezing of the data base. Baseline patient characteristics will be considered at the cluster (center) and patient levels. For the center level, characteristics at the beginning of the study will be described (there are no expected changes between the two periods for cluster characteristics). Baseline characteristics of patients will be described globally and according to the group of intervention. Continuous variables will be summarized using descriptive statistics, i.e., number of subjects, mean, median, standard deviation (SD), inter quartile range, minimum and maximum. Categorical variables will be summarized by frequency and percentage.

Since this is a non-inferiority study, analysis of the principal criterion will be performed on the per-protocol population (patients without major protocol deviation as no respect of selection criteria, no respect of strategy assigned by randomization, missing values for the principal criteria, or other major protocol deviation identified during data review before freezing of the data base). A sensitivity analysis will be performed on the intention-to-treat population (all randomized patients, except those that withdrew consent). Given the rare occurrence of a thrombo-embolic (TE) event, a generalized linear regression mixed model with Poisson distribution (log link) will be performed (Poisson model for proportions), taking into account a random effect for each center and considering period and strategy-by-period interactions as fixed effects. The decision rule will be based on the upper bound of the 95% two-sided CI of the incidence rates ratio of TE events. Any missing value will be considered as a failure. Secondly, sensitivity analysis will be performed using the 95% two-sided CI of the difference of percentage of TE events between groups. If the upper bound of the CI is above 1.35% of the difference, the inferiority hypothesis of the intervention group will be rejected. Secondary criteria will be compared between groups on the intention-to-treat population and under the superiority hypothesis. The proportions of irradiative imaging, of introduction of anticoagulation regimen, of hospital admission following the ED visit, of all causes of hospital readmission at 3 months, and of all-cause death at 3 months will be compared between groups by using generalized linear regression mixed models with Poisson distribution (log link), taking into account a random effect for each center and considering period and strategy-by-period interaction as fixed effects. If the number of events is sufficient, generalized linear regression mixed modeling with Bernoulli distribution (logit link) will be performed. The proportion difference between groups and its 95% CI will be calculated.

The ED length of stay will be compared between the two periods using a linear regression mixed model, taking into account a random effect for each center and considering period and strategy-by-period interaction as fixed effects. In case of non-normality distribution of the interest variable, a transformation could be performed or a model appropriate to data distribution could be selected.

Differences between groups in the proportion of patients with diagnosed PE at 3-month follow-up, excluding isolated sub-segmental PE, among patients in whom PE has been initially ruled out, will be calculated as well its 95% CI. If possible, generalized linear regression mixed models with Poisson distribution (log link) will be performed. A random effect for each cluster will be considered and considered fixed effects will be period and strategy-by-period interactions. If the number of events is sufficient, generalized linear regression mixed modeling with Bernoulli distribution (logit link) could be performed. The proportion of patients with a diagnosed TE event at 3-month follow-up (either a PE or a deep venous thrombosis), among patients in whom PE was initially ruled out by the PEPS score, will be described with its 95% CI.

Sensitivity analysis will be performed on the per-protocol population. Missing values for secondary criteria will not be replaced. All superiority tests will be two-sided and *p* values less than 0.05 will be considered significant.

### Medico-economic evaluation

This first economic evaluation of the innovative diagnostic strategy of PE follows the recommendations from the French national health authority and the CHEERS statement for single-trial-based studies [21].

Given the non-inferiority hypothesis, the primary analysis assumes that the modified diagnostic strategy is either dominant (cheaper and equally or more effective) or decrementally cost-effective (cost-reducing and possibly TE event increasing below the upper bound of the 95% two-sided CI of the incidence rate ratio of TE events) [22, 23]. The perspective of the analysis is the healthcare system and the time horizon is 3 months. Resources will be collected prospectively at the patient level using the study case report form supplemented by data from the hospital claims database for the entire duration of the study period. Costs for the index admission and in-trial follow-up period are assessed using a combination of resource-based and event-based methods. In-hospital resource utilization will be described based on diagnosis and procedural codes and length of stay for the entire duration of the index admission and subsequent hospital stays. Out of hospital resources will be estimated from the CRF and patient interviews during the follow-up visits. We will collect information on ED visits, consultations, medications, and imaging laboratory tests. Out of hospital resources will be valued using the latest price/tariff schedule. In view of the short duration of this study, costs and benefits are not discounted. The economic evaluation will use the difference in the safety of each strategy as the effectiveness criterion in accordance with previous studies [24]. The analysis is based on the entire population of patients included in the trial.

The unit of analysis is the patient. Costs will be presented as means with 2.5 to 97.5% bootstrapped intervals. Between-group comparisons of costs will be performed using the bootstrap *t*-test. A joint comparison of costs and effects will be performed by nonparametric bootstrapping with 1000 re-samples and the probabilities of better/worse performance of modified diagnostic algorithm and higher/lower costs will be determined.

### Ethics and dissemination

All eligible ED patients will be informed about the study by an information form in participating centers. The treating physician or the local investigator will explain the rationale and objectives of the study. An information note will be given to him/her, and he/she will be able to discuss the study with the physician or local investigator or research assistant. In France, since this is a cluster randomized trial with minimal risk and constraints, if a patient does provide freely-given oral consent, he/she can be included in the study. In addition, the investigator will specify in the person’s medical file the person’s participation in the research and the procedures for obtaining his/her oral consent. In Spain, signed informed consent from the patient will be sought.

This trial has been accepted by an institutional review board (IRB) in both France and Spain: Comité de protection des personnes Ile de France XI and Comité de Ética de la Investigación con medicamentos del Hospital Clínic de Barcelona.

Authorization from Agence Nationale de Sécurité du Médicament and Comité National Informatique et Liberté was also obtained before the start of the trial. There is no need of a data and safety monitoring board as this research is classified as “minimal risk”.

All modifications of the protocol will be shared with the relevant IRB for approval. The URC-Est clinical research platform and primary investigator will have access to the final dataset. Raw unidentified data can be shared for research purposes upon request after agreement with the sponsor and the primary investigator. The results of the trials and all potential ancillary or post-hoc analysis will be shared by communication in national congress and publication in peer review journals.

## Trial status

ClinicalTrials.gov Identifier: NCT04032769.

Recruitment status: Recruiting.

First posted: July 25, 2019.

Last update posted: November 19, 2019.

Study start date: October 1st 2019.

Expected study completion date: June 2020.

Protocol version: 2.1, 02/29/2020.

## Supplementary information

**Additional file 1.** Study protocol.

**Additional file 2.** Statistical analysis plan.

## Data Availability

A complete statistical analysis plan is available as an appendix. The trial dataset will be made available upon request to the authors or clinical research unit.
