# Supplementary material for: MOdified DIagnostic strateGy to safely ruLe-out pulmonary embolism In the emergency depArtment: study protocol for the Non-Inferiority MODIGLIANI cluster cross-over randomized trial
Source: Trials. 2020 Jun 3;21:458. doi: 10.1186/s13063-020-04379-y (PMC7268276; doi:10.1186/s13063-020-04379-y)
Supplement: Supplementary file 1 — Additional file 1. Study protocol. [file 13063_2020_4379_MOESM1_ESM.pdf]

MOdified DIagnostic strateGy to safely ruLe-out pulmonary embolism In the emergency depArtment: A Non-Inferiority cluster cross-over randomized trial
(MODIGLIA-NI)

RESEARCH PROTOCOL INVOLVING HUMAN PARTICIPANTS WITH MINIMAL RISKS AND BURDEN

Version N°2.1 of 29/02/2019
Protocol code number: APHP180595 / IDRCB no. 2019-A00553-54

Coordinating Investigator:	Yonathan FREUND<br>Emergency department,<br>Hôpital Pitié-Salpêtrière, AP-HP, Paris France<br>Tel: +33663549017 – mail: yonathan.freund@gmail.com
Sponsor:	ASSISTANCE PUBLIQUE - HOPITAUX DE PARIS (AP-HP)<br>and by delegation: Clinical Research and Innovation Department (DRCI)<br>Hôpital Saint-Louis<br>1, avenue Claude Vellefaux<br>DRCI head office project advisor: Sophie Courtial-Destembert<br>Email sophie.courtial-destembert@aphp.fr

Entity responsible for monitoring the study data management and statistical analysis:
Unité de Recherche Clinique du GH HUEP (URC-Est)
184 rue du Faubourg Saint Antoine, Paris 12ème
DRCI-URC (Clinical Research Unit) project advisor: Sarah Sahli
Email: saranesrine.salhi@aphp.fr

Clinical Research and Innovation Department (DRCI)
Hôpital Saint Louis 75010 PARIS

Economic evaluation	URCEco<br>Pr. Isabelle Durand-Zaleski (PU-PH)<br>ECEVE, UMRS 1123<br>URCEco Ile de France, Hôpital de l'Hôtel Dieu<br>1 place du parvis de Notre Dame<br>75004 Paris France

This document is the property of DRCI / AP-HP. All reproduction is strictly prohibited.

PROTOCOL SIGNATURE PAGE

Research code number: APHP180595

Title: MOdified DIagnostic strateGy to safely ruLe-out pulmonary embolism In the emergency depArtment: A Non-Inferiority cluster cross-over randomized trial (MODIGLIA-NI)

Version N°2 of: 05/12/2019

The study will be carried out in accordance with the protocol, with current good practices and with statutory and regulatory requirements.

**Coordinating Investigator**:<br>Yonathan FREUND<br>Emergency department,<br>Hôpital Pitié-Salpêtrière, AP-HP,<br>Paris<br>France	Date: ……………/………/……….. Signature:
**Sponsor**<br>Assistance Publique-Hôpitaux de Paris<br>Clinical Research and Innovation Delegation (DRCI)<br>Hôpital Saint Louis<br>1 avenue Claude Vellefaux<br>75010 Paris	Date: ……………/………/……….. Signature:

This document is the property of DRCI / AP-HP. All reproduction is strictly prohibited.

TABLE OF CONTENTS

1 SUMMARY 5

2 SCIENTIFIC JUSTIFICATION FOR THE RESEARCH 8

2.1 CURRENT KNOWLEDGE 8
2.2 HYPOTHESES FOR THE RESEARCH 10
2.3 DESCRIPTION OF THE POPULATION OF RESEARCH PARTICIPANTS AND JUSTIFICATION FOR THE CHOICE OF PARTICIPANTS 10
2.4 INTERVENTIONS AND PRODUCTS WHICH WILL BE PERFORMED OR USED AS STANDARD 10
2.5 INTERVENTIONS ADDED FOR THE RESEARCH 10
2.6 SUMMARY OF THE KNOWN AND FORESEEABLE BENEFITS AND RISKS FOR THE PARTICIPANTS 11

3 OBJECTIVES OF THE RESEARCH 12

3.1 MAIN OBJECTIVE OF THE RESEARCH 12
3.2 SECONDARY OBJECTIVES 12

4 DESCRIPTION OF THE RESEARCH 12

4.1 PRIMARY ENDPOINT 12
4.2 SECONDARY ENDPOINTS 12

5 RESEARCH METHODOLOGY 13

5.1 DESIGN OF THE STUDY 13
5.2 NUMBER OF PARTICIPATING SITES 13
5.3 AVOIDING AND REDUCING BIAS 13

6 PROCEDURE FOR THE RESEARCH 14

6.1 SCHEDULE FOR THE STUDY 14
6.2 TABLE SUMMARIZING THE CHRONOLOGY OF THE RESEARCH 15
6.3 DISTINCTION BETWEEN STANDARD CARE AND RESEARCH 15

7 ELIGIBILITY CRITERIA 15

7.1 INCLUSION CRITERIA 15
7.2 NON-INCLUSION CRITERIA 16
7.3 ENROLMENT PROCEDURE 16

8 TERMINATION AND EXIT RULES 17

8.1 CRITERIA AND PROCEDURE FOR PREMATURE WITHDRAWALS AND EXITS FROM THE STUDY 17

9 EFFICACY ASSESSMENT 18

9.1 DESCRIPTION OF PARAMETERS FOR ASSESSING EFFICACY ENDPOINTS 18
9.2 ANTICIPATED METHODS AND TIMETABLE FOR MEASURING, COLLECTING AND ANALYZING THE EFFICACY DATA 18

10 SAFETY 18

11 SPECIFIC COMMITTEES FOR THE STUDY 19

11.1 STEERING COMMITTEE 19
11.2 ENDPOINT ADJUDICATION COMMITTEE 19

12 DATA MANAGEMENT 19

12.1 DATA COLLECTION 19
12.2 IDENTIFICATION OF DATA RECORDED DIRECTLY IN THE CRF WHICH WILL BE CONSIDERED AS SOURCE DATA 19
12.3 RIGHT TO ACCESS SOURCE DATA AND DOCUMENTS 20
12.4 DATA PROCESSING AND STORAGE OF RESEARCH DOCUMENTS AND DATA 20
12.5 OWNERSHIP OF THE DATA 21

13 STATISTICAL ASPECTS 21

13.1 DESCRIPTION OF STATISTICAL METHODS TO BE USED INCLUDING THE TIMETABLE FOR THE PLANNED INTERIM ANALYSES 21
13.2 CALCULATION HYPOTHESES FOR THE NUMBER OF SUBJECTS REQUIRED AND THE RESULT 22
13.3 ANTICIPATED LEVEL OF STATISTICAL SIGNIFICANCE 23
13.4 STATISTICAL CRITERIA FOR TERMINATION OF THE STUDY 23
13.5 METHOD FOR TAKING INTO ACCOUNT MISSING, UNUSED OR INVALID DATA 23
13.6 MANAGEMENT OF MODIFICATIONS MADE TO THE ANALYSIS PLAN FOR THE INITIAL STRATEGY 23
13.7 CHOICE OF INDIVIDUALS TO INCLUDE IN THE ANALYSES 23

This document is the property of DRCI / AP-HP. All reproduction is strictly prohibited.

13.8 MEDICO-ECONOMIC EVALUATION 23
14 QUALITY CONTROL AND ASSURANCE 25
14.1 GENERAL ORGANIZATION 25
14.2 CASE REPORT FORMS 25
14.3 MANAGEMENT OF NON-COMPLIANCES 25
14.4 AUDITS 25
14.5 PRINCIPAL INVESTIGATOR'S DECLARATION OF RESPONSIBILITY 26
15 ETHICAL AND LEGAL CONSIDERATIONS 26
15.1 METHODS FOR INFORMING THE RESEARCH PARTICIPANTS 26
15.2 PROHIBITION OF CONCOMITANT CLINICAL STUDIES PARTICIPATION AND EXCLUSION PERIOD AFTER THE STUDY 26
15.3 COMPENSATION FOR PARTICIPANTS 26
15.4 LEGAL OBLIGATIONS 26
15.5 REQUEST FOR APPROVAL FROM THE CPP 27
15.6 INFORMING THE ANSM 27
15.7 DECLARATION OF COMPLIANCE WITH THE MR-003 "REFERENCE METHOD" 27
15.8 MODIFICATIONS TO THE STUDY 27
16 FUNDING AND INSURANCE 27
16.1 SOURCES OF MONETARY SUPPORT 27
16.2 INSURANCE 27
17 PUBLICATION RULES 28
17.1 MENTION OF AP-HP AFFILIATION FOR PROJECTS SPONSORED BY AP-HP 28
17.2 MENTION OF THE SPONSOR AP-HP (DRCI) IN THE ACKNOWLEDGEMENTS OF THE TEXT 28
17.3 MENTION OF THE FUNDER IN THE ACKNOWLEDGEMENTS OF THE TEXT 28
17.4 AUTHOR LISTS 28
18 REFERENCES 28
19 ADDENDA 32
19.1 LIST OF INVESTIGATORS 33

This document is the property of DRCI / AP-HP. All reproduction is strictly prohibited.

1 SUMMARY

Full title	MOdified DIagnostic strateGy to safely ruLe-out pulmonary embolism In the emergency depArtment: A Non-Inferiority cluster cross-over randomized trial
Acronym	MODIGLIA-NI
Coordinating Investigator	Yonathan FREUND
Sponsor	Assistance Publique-Hôpitaux de Paris
Scientific justification	The diagnosis of Pulmonary Embolism (PE) is a crucial matter in the Emergency Department (ED). The overall prevalence of PE in suspected patients continue to decrease, and the rate of diagnostic failure is now below 1% in Europe and in the USA.Because a missed PE could be potentially lethal, several researchs reported that PE is both overinvestigated and overdiagnosed. The diagnostic gold standard for PE is the computed tomographic pulmonary angiogram (CTPA) and has been shown to have clear risks (allergic reaction, acute renal failure, delayed solid tumor) and other downsides such as prolonged ED stay and increased cost. To limit the use of CTPA, two rules were recently reported to be safe to exclude PE: the PERC rule and the YEARS rule. PERC is an 8 item block of clinical criteria that has recently been validated to safely exclude PE in low risk patients. YEARS is a clinical rule that allow to raise the threshold of D-dimer for the order of CTPA. However, whether a modified diagnostic algorithm that includes these two rules combined could safely reduce imaging study use in the ED is unknown.
Main objective and primary endpoint	The primary objective of this trial is to assess the safety of a modified diagnostic strategy (MODS) with theYEARS for patients in whom PE was not excluded by PERC score in the ED.<br><br>The primary endpoint is the failure percentage of the diagnostic strategy, defined as a diagnosed thrombo-embolic event at 3 month follow-up (either a PE or a deep venous thrombosis), among patients in whom PE has been initially ruled out.
Secondary objectives and endpoints	To assess the efficacy of the modified diagnostic strategy (MODS) in reducing order of irradiative imaging studies, ED length of stay, undue onset of anticoagulation regimen, hospital admission, hospital readmission, and mortality at 3 months.<br>To evaluate the efficacy of the modified diagnostic strategy to reduce overall 3-months total cost.<br>To test the safety of the PEPS score.<br><br>Secondary endpoints include:<br>- CTPA or V/Q scan<br>- Anticoagulant therapy administration<br>- Length of stay in the ED (hours)<br>-Admission to the hospital following ED visit.<br>- All causes re hospitalization at 3 months,<br>- Death from all causes at 3 months<br>-Diagnosed pulmonary embolism at 3 month follow-up excluding the isolated sub-segmental pulmonary embolism, among patients in whom PE has been initially ruled out.

This document is the property of DRCI / AP-HP. All reproduction is strictly prohibited.

	- PEPS score<br>- 3 months total cost and cost effectiveness
Design of the study	This is a non-inferiority, cluster cross-over randomized, international trial.<br>Each center will be randomized on the sequence of periods: intervention (MOdified Diagnostic Strategy: MODS) followed by control (usual care), or control followed intervention with 1 month of "wash-out" between the two periods.
Population of study participants	Adult emergency patients, with a suspicion of PE
Inclusion criteria	Any symptom from:<br>- Acute onset of, or worsening of dyspnea<br>- Chest pain<br>- Syncope<br>Free given Oral consent
Exclusion criteria	- Other obvious cause than PE for chest pain, syncope or dyspnea<br>- High clinical probability of PE (estimated by the physician gestalt as > 50%)<br>- Low clinical probability of PE (estimated by the physician gestalt as <10%) and PERC negative patients<br>- Acute severe presentation (clinical signs of respiratory distress, hypotension, SpO2<90%, shock)<br>- Concurrent anticoagulation treatment<br>- Current diagnosed thrombo-embolic event<br>- Prisoners<br>- Pregnancy<br>- No social security<br>- Participation in another intervention trial<br>- Anticipated inability to follow up at 3 month
Interventions under investigation	Modified diagnostic strategy (MODS):<br>All included patients will be tested with quantitative D-dimer. The MODS work-up will be based on YEARS rule, that included three criteria (hemoptysis, signs of DVT, PE is the most likely diagnosis)<br>- If all YEARS criteria are absent, the threshold of D-Dimer for ordering a CTPA will be raised at 1000 ng/ml.<br>- If at least one criterion of YEARS is present, then the D-dimer threshold for ordering a CTPA will be as usual (500 ng/ml, or agex10 for patients aged 50 and over)
Comparator arm	All included patients will be tested with D-Dimer, the threshold for ordering a CTPA will be as usual (conventional age-adjusted threshold at 500 ng/ml, or agex10 for patients aged 50 and over).
Expected benefits for the participants and for society	Pulmonary Embolism is a diagnosis that concerns nearly 200 000 patients each year in France. The multiplication of diagnostic studies led to a rise in PE diagnosis, associated with a concurrent rise in the diagnosis of less severe PE, and no subsequent decrease in mortality. The prevalence of PE among patients with a non-high clinical probability is around 10%. This low prevalence translates an overexposition of patients to harm mainly from radiation exposure, intravenous contrast administration (allergic reaction and acute renal failure), prolonged length of stay in the ED and anticoagulation treatment initiation. Besides these reports of PE overtesting, evidences of PE overdiagnosing emerged, which is caused both by the

This document is the property of DRCI / AP-HP. All reproduction is strictly prohibited.

	diagnosis of small PEs in which equipoise remains on the anticoagulation treatment indication, and by the inevitable false positive cases of CTPA that would expose patients to undue anticoagulation treatment and prolonged length of hospital stay.<br>A post-hoc analysis of two recent large cohorts in France and Belgium (PROPER and PERCEPIC) showed that, in low risk patients, the endorsement of the combination of PERC then YEARS rules would have resulted in a 50% reduction of CTPA use, and a failure rate of 0.83% (95% CI 0.51 – 1.35), which is acceptable as defined by the International Society of Thrombosis and Haemostasis, (i.e. < 1.85%).<br>Safely reducing the use of CTPA would be beneficial for the patients, by limiting their risk of associated adverse events and overdiagnosis of PE, and will also reduce their length of stay in the ED, which is associated with better outcomes. Furthermore, reducing supplemental investigations for patients with suspicion of PE may also reduce the cost of ED visits, which would be of great benefit in the context of increasingly resource stretched healthcare services.<br>If our hypothesis is demonstrated, the results could change practice by modifying the ED diagnostic strategy.
Minimal risks and constraints added by the study	Patients in the intervention group will be exposed to a risk of diagnostic failure and missed PE. This risk has been recently evaluated by our team and remains low (0.83%, as defined by the International Society of Thrombosis and Haemostasis, being < 1.85%).
Scope of the study	Emergency department
Number of participants included	1400
Number of sites	- 16 Emergency Departments in France<br>- 04 emergency departments in Spain
Schedule for the study	- Inclusion period: 2 periods of maximum 4 months with a wash-out period of 1 month between the 2 recruitment periods<br>- Participation period (treatment+follow-up): 3 months<br>- Total duration of the study: 12 months
Number of enrolments expected per site and per month	8,75
Statistical analysis	No interim analysis is planned. Analysis will be performed at the end of the study after data review and freezing of data base according to Per Protocol principle and with regard to cluster level randomisation.
Sources of monetary support	French ministry of health

This document is the property of DRCI / AP-HP. All reproduction is strictly prohibited.

2 SCIENTIFIC JUSTIFICATION FOR THE RESEARCH

2.1 CURRENT KNOWLEDGE

2.1.1 About the condition under investigation

The incidence of pulmonary embolism (PE) in France and Europe has been estimated to 0.6-0.9 per 1000 persons per year.[1,2]PE is a potential lethal diagnosis,[3] and its diagnosis in the Emergency Department (ED) is challenging.[4] The fear of missing this diagnosis and the poor specificity of its clinical presentation lead physicians to suspect PE in all patients who present with a broad variety of symptoms such as dyspnea, chest pain, syncope, and hypotension. This represents a volume of ED patients of more than 10 million a year in the United States. The standard strategy of the last decade for PE diagnosis is as presented in figure 1.

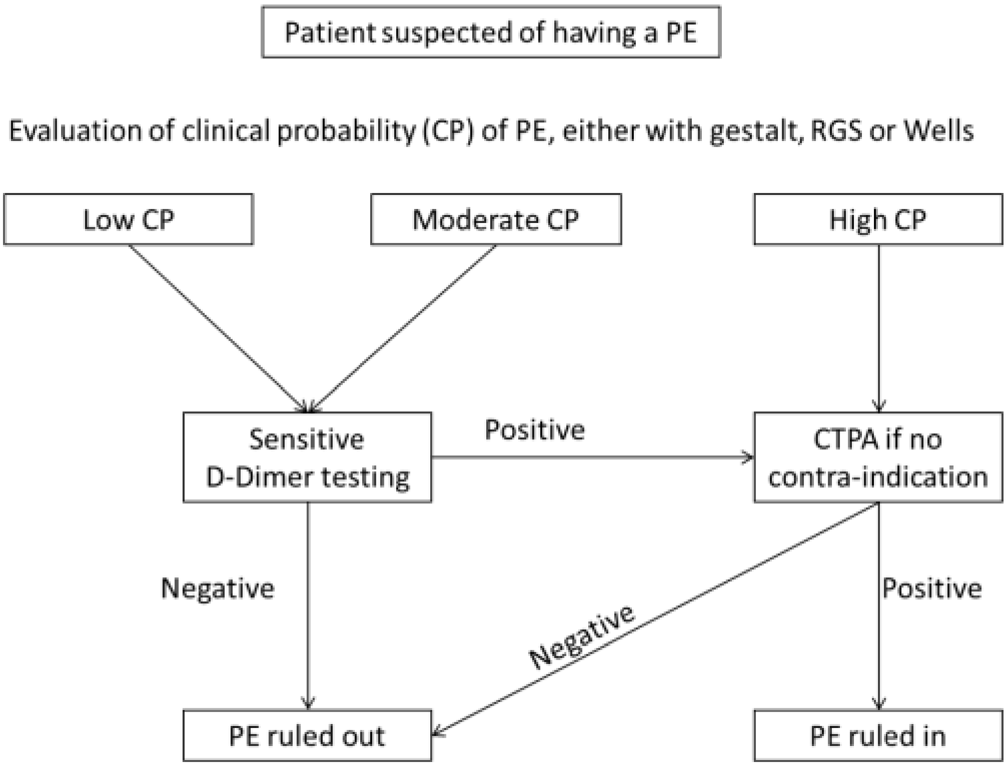

Figure 1: Standard strategy: recommended diagnosis work up for PE in the emergency department. PE: Pulmonary Embolism, RGS: Revised Geneva Score, CTPA: Computed Tomography of Pulmonary Artery.[5]

The usual work up for PE diagnosis first includes an assessment of clinical probability of having a PE. This assessment can be made using a structured score (Revised Geneva Score RGS or Wells score),[6,7] or an unstructured estimation of the clinical probability (referred to as the clinician “gestalt”)[8–10]. Then a sensitive D-dimer testing is performed in patient with low to moderate clinical probability, followed if positive by a Computed Tomography Pulmonary Angiogram (CTPA) in the absence of contra-indication. Patients with a high clinical probability should undergo CTPA without the need for preliminary testing. This diagnosis strategy is recommended by European guidelines [5], national expert recommendations [11] and local policies. It has been validated and is safe to exclude PE in outpatient visiting ED.[12] However, due to its low specificity (40-60%)[10,13] D-dimer testing may lead to more than 50% of false positive and subsequent CTPA.[10] Furthermore, the wide availability of D-dimer testing combined to the fear of missing a PE led to a lowering in the threshold for testing patients for PE, hence the decrease in the prevalence of confirmed PE amongst suspected patients from 30% to around 10%.[14–19]
Subsequently, in the last 15 years, there has been a marked rise (up to 15 fold) in the utilization of CTPA [20] and in the incidence of diagnosed pulmonary embolism (of about 2 fold in 10 years).[21] However, this greater incidence of PE was not followed by a decrease in mortality from PE, but

This document is the property of DRCI / AP-HP. All reproduction is strictly prohibited.

rather a decrease in PE fatality.[21,22] This suggests that PE tends to be “overdiagnosed”: small PEs are more frequently diagnosed, with no clear benefit in terms of outcomes. This increased exposure to CTPA may be a source of unnecessary risks, such as contrast-induced nephropathy (4-12% of patients[23]) and allergic reactions, adverse events after anticoagulation treatment or the delayed occurrence of radiation-induced cancer (extrapolated to roughly 1/1000).[23–25]

2.1.2 Maximal acceptable failure rate of a PE diagnostic strategy in the ED

In the last decades, a diagnostic strategy was considered safe to rule-out PE if it was associated with a failure rate of less than 3%. This was derived from old data of the negative predictive value (NPV) of the then-gold standard pulmonary angiography. The pivot trials that constitute the basis of our guidelines were built under the hypothesis of a maximal failure rate of 3%.[12,13,19,26–28]
However, the scientific subcommittee of the international society of thrombosis and haemostasis (ISTH) recently published a recommendation on the maximal rate that should be considered acceptable when testing a new diagnostic strategy.[29] As the authors recommend, this should be considered with regards to the specific PE prevalence of the studied population. Therefore, the authors recommend that this maximal acceptable failure rate should be 1.82+0.005*prevalence.[29]
With a reported PE prevalence between 4% and 14%, **a conservative approach would set this maximal acceptable failure rate at 1.85%.**[16,18,30]

2.1.3 The PERC and YEARS rules

To reduce the rate of unnecessary testing for PE caused by overuse of D-dimer, Kline et al. developed in 2004 a block rule of eight binary variables (PERC rule, cf Table 1): age <50 years, pulse <100bpm, SaO2 >94%, no unilateral leg swelling, no hemoptysis, no recent trauma or surgery, no prior PE or deep venous thrombosis and no exogen estrogen use [15] – PERC negative patients are defined as fulfilling these 8 criteria.
After a few controversies, this rule has been validated in both US and European population, with a reported failure rate of a PERC based strategy of below the maximal acceptable failure rate of 1.85%.[17,19,30] Therefore, we will consider patients suspect of PE only if they have at least one positive item of the PERC.

**Age > 50**	**Yes/No**
**Heart rate > 100**	**Yes/No**
**SaO2 < 95%**	**Yes/No**
**Unilateral leg swelling**	**Yes/No**
**Hemoptysis**	**Yes/No**
**Recent trauma or surgery**	**Yes/No**
**History of PE or DVT**	**Yes/No**
**Exogenestrogen use**	**Yes/No**

If at least one item of the PERC is “yes”, the score will be considered as positive.

If all items of the PERC are “No”, the score will be considered as negative, and the patient will not be included (Cf. Non inclusion criteria)

Table 1: *Items of the Pulmonary Embolism Rule-out Criteria (PERC). PE: pulmonary embolism. DVT: deep venous thrombosis*

Recently, the YEARS rule was studied to this same goal of reducing the rate of CTPA. This rule allowed the clinicians to raise the conventional D-dimer threshold (500 ng/ml, or agex10 for patients aged 50 and over) to 1000 ng/ml when the three clinical criteria of the YEARS rule are absent (cf table 2) (hemoptysis, signs of DVT, PE is the most likely diagnosis). Two recent prospective multicentre cohort studies (although not comparative) confirmed that this rule was safe (i.e. failure rate below <1.85%).[1631] However these studies only assessed the YEARS rule, while PERC was not applied to their patients. This could be a major shortcoming as the added value and the safety of one rule over the other has not been prospectively evaluated.

This document is the property of DRCI / AP-HP. All reproduction is strictly prohibited.

**Clinical signs or symptoms of DVT**	**Yes/No**
**Hemoptysis**	**Yes/No**
**PE is the most likely diagnosis**	**Yes/No**

If at least one item of the YEARS is "yes", the score will be considered as positive.

Table 2: *YEARS item. DVT: deep venous thrombosis*

Both PERC and YEARS have been reported to reduce the number of CTPA in the ED, and therefore these two rules should be endorsed in the routine practice. However, it is unknown whether these two rules could be safely combined. To date, no prospective study evaluated the safety of YEARS applied only in PERC positive patients.

We recently published a post-hoc analysis of the two large recent cohorts of patients with suspicion of PE and found that these two rules could be safely combined with a failure rate of 0.83% [95% CI 0.51 - 1.35]. This combination would have allowed a relative reduction of almost 50% of CTPA in the ED.[32]

2.2 Hypotheses for the research

We hypothesize that a modified diagnostic strategy (**MODS**) to rule-out PE in the ED, which combines the PERC and YEARS rules, would be associated with a failure rate below 1.85%.

2.3 Description of the population of research participants and justification for the choice of participants

This study will include patients with a suspicion of PE defined as:

1) - acute onset of new or worsening shortness of breath
- or chest pain,
- or syncope

in the absence of any obvious other cause (such as pneumothorax, asthma attack, ST elevation myocardial infarction, trauma, etc.)

We will exclude patients with a high clinical probability estimated by the clinician gestalt.
Since patients with a high clinical probability should be investigated with CTPA or V/Q scan, our modified algorithm will not concern them so we will not include them.
As described in previous studies, [15,33] *this assessment is established as the answer of the question "How do you estimate the pre-test clinical probability: low, moderate, or high?* [10]

We will also exclude patients with a low clinical probability and a PERC score = 0, as it has been validated that these patients have a very low risk of PE and should not been investigated for PE.

2.4 Interventions and products which will be performed or used as standard

In the control period, work-up for PE will be conducted as recommended for patient with non-high clinical probability:
Test with quantitative D-dimer, followed if positive by a CTPA (or V/Q scan if CTPA is contra-indicated). <u>A positive D-dimer is defined as >500 ng/ml, or >agex10 for patients aged 50 and over.</u>

2.5 Interventions added for the research

In the intervention period, work-up for PE will also first include a D-dimer test and will follow the **MODS strategy**:

This document is the property of DRCI / AP-HP. All reproduction is strictly prohibited.

All patients will be assessed with the YEARS rule by the emergency physicians:

- in the absence of hemoptysis, clinical sign of deep venous thrombosis and if PE is not the most likely diagnosis, then the threshold for positive D-dimer will be raised at 1000 ng/ml.
- If any of the YEARS items is positive, then the patient will follow standard work-up with the conventional D-dimer threshold (i.e. as >500 ng/ml, or >agex10 for patients aged 50 and over).

2.6 Summary of the known and foreseeable benefits and risks for the participants

Pulmonary Embolism is a diagnosis that concerns nearly 200 000 patients each year in France. The multiplication of diagnostic studies led to a rise in PE diagnosis, associated with a concurrent rise in the diagnosis of less severe PE, and no subsequent decrease in mortality [20–22].

Several studies reported that PERC or YEARS can be safely endorsed in the ED, with a subsequent absolute reduction of roughly 10-15% of CTPA for each rule alone. We recently performed a post-hoc analysis and showed that these two rules could be safely combined, with a low risk of diagnostic failure at 0.83% (95%CI 0.51 to 1.35) therefore <1.85% as defined safe by the ISTH.

Furthermore, because of our exclusion criteria (acute presentation, high clinical probability) potential false negative patients would belong to the group of PE of lower risk, with an estimated 30 days mortality below 1% [34–36]. With an anticipated diagnostic failure percentage below 1.85%, the overall extrapolated added risk would be rare, below 1/5 000 at 30 days in the experimental group.

This modified algorithm that would include this combination could help to further reduce the percentage of irradiative imaging studies. Such reduction in imaging studies would be beneficial for patients. Newman and Schriger extrapolated that the potential harm resulting from the three major risks of further testing outweighs the benefit a group of patients with a PE prevalence below 1.85%[37] The main medical harms that can be caused by unnecessary testing for PE include adverse events from CTPA (allergic reaction and acute renal failure), delayed solid tumor increased risk from irradiative imaging, and iatrogenic complication of anticoagulation for positive tested patients (either false or true positive).

Besides thisdirect risk for the patient, further testing has clear downsides: a prolonged stay in the ED, with potential subsequent overcrowding [38,39] , overall worse short term outcomes [40], and increased cost. In the PROPER trial we showed that this reduction in resource use was directly associated with a significant reduction in ED length of stay (a mean reduction of 36min, i.e. relative reduction of more than 10%).[30]

Finally, avoiding any supplemental investigations for patients with a suspicion of PE may also reduce the cost of ED visits, which would be of great benefit in the context of increasingly resource stretched healthcare services. Thus, in case of the demonstration of non-inferiority, the modified diagnostic strategy will safely and substantially reduce the volume of imaging studies, and therefore irradiation, adverse events, length of ED stay and overcrowding.

If our hypothesis is demonstrated, the results could change practice by modifying the ED diagnostic strategy, limiting the risk of overtesting and overdiagnosing PE, impacting health expenditures, and improving workflow in the ED with subsequent potential benefit for any ED patient.

This document is the property of DRCI / AP-HP. All reproduction is strictly prohibited.

3 OBJECTIVES OF THE RESEARCH

3.1 Main objective of the research

The primary objective of this trial is to assess the safety of a modified diagnostic strategy (MODS) with the YEARS rulefor patients in whom PE was not excluded by PERCscore in the ED.

3.2 Secondary objectives

To assess the efficacy of the modified diagnostic strategy in reducing:

- order of irradiative imaging studies,
- undue onset of anticoagulation regimen,
- ED length of stay,
- hospital admission,
- hospital readmission and mortality at 3 months.

To assess the safety of a modified diagnostic strategy (MODS) with the YEARS rule for patients in whom PE was not excluded by PERC score in the ED on diagnosed pulmonary embolism at 3 month follow-up excluding the isolated sub-segmental pulmonary embolism, among patients in whom PE has been initially ruled out.

To assess the safety of the PEPS score.

To evaluate the efficacy of the modified diagnostic strategy to reduce overall 3-months total cost and cost effectiveness (cost per major adverse event averted, namely hospitalisation, rehospitalisation, imaging study, death).

4 DESCRIPTION OF THE RESEARCH

4.1 Primary endpoint

The primary endpoint is the failure percentage of the diagnostic strategy, defined as a diagnosed thrombo-embolic event at 3 month follow-up (either a PE or a deep venous thrombosis), among patients in whom PE has been initially ruled out.

Exclusion of PE in the ED is made upon negative D-dimer result or negative CTPA or V/Q scan in both groups.
Follow up at 3 months will be made upon telephone interview of the patient or his general practitioner, outpatient consultation or email or hospital visit. The primary criterion of thrombo-embolic event will be based on an objective diagnosis of DVT on Doppler ultrasonography, an intraluminal defect on CTPA, or a V/Q lung scan with a reported high probability. To confirm the occurrence of the primary endpoint, all files with evidence of thrombo-embolic event collected by the local investigator of each center will be independently reviewed by an adjudication committee of three experts, blinded one to each other, and blinded to the study period. The adjudication committee will also review cases of death with no evidence of thrombo-embolic event and will adjudicate the death as likely related to a PE or not. A sudden death in the absence of other obvious cause will be adjudicated as related to a PE.

4.2 Secondary endpoints

Secondary endpoints include:

- CTPA or V/Q scan
- Anticoagulant therapy administration
- Length of stay in the ED (hours)
- Admission to the hospital following ED visit,
- All causes re hospitalization at 3 month,
- Death from all causes at 3 month.
- Diagnosed pulmonary embolism at 3 month follow-up excluding the isolated sub-segmental pulmonary embolism, among patients in whom PE has been initially ruled out.
- PEPS score
- 3 month total cost and cost effectiveness (cost per major adverse event averted).

This document is the property of DRCI / AP-HP. All reproduction is strictly prohibited.

5 RESEARCH METHODOLOGY

5.1 Design of the study

This is a prospective multicenter, cluster randomized, non-inferiority controlled study.
Each center will be randomized on the sequence of periods: intervention (MOdified Diagnostic Strategy: MODS)followed by control(usual care), or control followed intervention with 1 month of "wash-out" between the two periods.The participating centers will implement the first assigned strategy until a target number of consecutive patients will be reached in total (half of the patients to be included in total, namely a minimum of 350 patients in each strategy); then in the cross-over phase, the centers will implement the second assigned strategy in a similar number of consecutive patients. The reporting of this study will follow the CONSORT statement extended to cluster randomized trial [41].

Justification for cluster randomization
In this study, cluster randomization design was preferred to an individual randomization (RCT- randomized controlled trial) to enhance the feasibility of the study by all the centers, in particular regarding the recruitment. Eligible patients are suspected to have a pulmonary embolism (i.e. a potentially life threatening condition). In this emergency setting, we strongly believe that centralized randomization at patient level is likely to make patients inclusion less feasible. In a busy emergency department, the individual randomization can be seen as an obstacle and a curb to the ED workflow. This would have preclude to the inclusion of truly consecutive patients, whilst as we experienced it, a cluster design allows a fast recruitment and will limit inclusion bias.[30]

The two periods will include a similar work-up strategy for PE that first mandates a D-dimer testing, and will differ by the threshold of D-dimer to rule-out PE or order further imaging study:

Control period:
During the control period, the threshold of D-dimer will be as usual, being “>500 ng/ml” for patients aged less than 50 and “> agex10” for patients aged 50 and over.
A positive result of D-dimer and the absence of other obvious cause for PE will mandate a CTPA, or V/Q scan if CTPA is contra-indicated.
A negative result of D-dimer will rule out PE.

Intervention period (MODS):
During the intervention period the threshold of D-dimer will depend on the YEARS rule (MODS stragegy):

1) If all the three items of YEARS are negative (i.e. No hemoptysis, No clinical sign of deep venous thrombosis and PE is not the most likely diagnosis), then the threshold of D-dimer will be raised at 1000 ng/ml.
2) If at least one item of YEARS is positive, then the threshold will remain unchanged (“>500 ng/ml” for patients aged < 50 and “> agex10” for patients aged 50 and over).
*A positive result of D-dimer and the absence of other obvious cause for PE will mandate a CTPA, or V/Q scan if CTPA is contra-indicated.
*A negative result of D-dimer will rule out PE.

5.2 Number of participating sites

20 Emergency Departments in France.

04 Emergency Departments in Spain.

5.3 Avoiding and reducing bias

5.3.1 Participant identification

The participants in this research will be identified as follows:

This document is the property of DRCI / AP-HP. All reproduction is strictly prohibited.

Site number (3 digits) - Sequential enrolment number for the site (4 digits) - surname initial - first name initial
This reference number is unique and will be used for the entire duration of the study.

5.3.2 Randomization

Sites sequence randomization will be computer generated by a biostatistician from URC-Est, independent of the study and before the study beginning. Randomization will be stratified on country and center size.

6 PROCEDURE FOR THE RESEARCH

For the study period, patients that attend to one of the participating center will be screened for eligibility by emergency physicians and research assistant. All ED patients will be informed of the study by an information form in participating centers.
When a patient is screened eligible, an information note will be given to him. If the patient does express his oral consent in France or written consent in Spain, he can be included in the study.

Whose oral consent must be obtained	Who informs the individuals and collects their oral consent	At what point the individuals are informed
The patient	Local investigator or the emergency physician in charge	In the ED, before the start of PE work up

6.1 Schedule for the study

- Enrolment period: 2 periods of maximum 4 months with a wash-out period of 1 month between the 2 recruitment periods.
- Duration of participation by each patient: 3 months
- Total study duration: 12 months
- Point of randomization: before the study start, centers will be randomized for the study period order.

6.1.1 Screening visit and baseline visit

All patients with chest pain, dyspnea or syncope will be screened and included in the ED by emergency physicians and research assistant. If the treating emergency physician or local investigator considers that the patient has a sufficient clinical suspicion of PE that he needs formal work up for this diagnosis, and that this suspicion is low enough to discard this suspicion in case of negative D-dimer (i.e. a non-high clinical probability), then the patient will be eligible.
The emergency physician or local investigator will confirm the presence of inclusion criteria and absence of any exclusion criteria.
Following the patient information and consent, the patient will be included in the study. Since all eligible patients will undergo a D-dimer test and that the MODS strategy will only influence the threshold of D-dimer.

6.1.2 Follow-up visits

Included patients will be followed up by phone interview or an email or a hospital visit at three months (13 weeks) by the local investigator with the help of a clinical research technician. The time frame of three months could be subject to minor adjustment, and will occur between day 84 and day 98. Follow up visit or interview will seek the occurrence of thrombo-embolic event (DVT documented with ultrasonography of the lower limbs or venous CT, or PE documented with positive CTPA or high probability V/Q lung scan), death, hospitalisation,to found report of thrombo-embolic event, or anticoagulation. In cases of death, or report of a thrombo-embolic event, the file will be analysed by a committee of three independent experts.

This document is the property of DRCI / AP-HP. All reproduction is strictly prohibited.

If the patient cannot be contacted, a next of kin or family member will be contacted. If not possible, the family physician will be contacted. If follow up is impossible, the investigators or the clinical research technician will contact the city hall and administrative service of his hometown to seek for possible death.

This method of adjudication has been described and validated in all major previous diagnostic studies on PE. [26,30,42]

6.1.3 Chronology of the research and end date

Each center will have 2 periods of recruitment of maximum 4 months. A wash-out period of 1month is planned between the two recruitment periods. Thus total inclusion period will last 8months. Patient duration of follow-up will be 3 months. The total duration of the study will be 12 months. Duration of each recruitment period in each center could be shorter, as each center will close recruitment once the target is reached to ensure similar recruitment in the two periods.

6.2 Table summarizing the chronology of the research

**Actions**	**Day 0 (Inclusion visit)**	**Month 3**
Information and consent of the patient	X	
History	X	
Clinical exam	X	
Para-clinical exam<br>(D-dimer +/- CTPA if indicated)	X	
YEARS score assessment	X in the MODS period	
Adverse events<br>(including thrombo-embolic event)	X	X

6.3 Distinction between standard care and research

TABLE: "Standard care" vs. "Added interventions" required specifically for the research

**Procedures and treatments carried out as part of the research**	**Procedures and treatments associated with care**	**Procedures added because of the research**
YEARS clinical assessment*		X
Follow-up by phone or email or hospital visit at 3 months		X
D-dimer testing	X	
Modification of D-dimer threshold		X
CTPA if D-dimer positive	X	

*: YEARS assessment corresponds to the responses of the 3 items of the YEARS rule by a clinical evaluation of the emergency physician, as reported in table 2.

7 ELIGIBILITY CRITERIA

7.1 Inclusion criteria

Patient aged ≥ 18 years that presents to an ED
With new onset of or worsening of shortness of breath or chest pain or syncope
Free given Oral consent expressed by the patient

This document is the property of DRCI / AP-HP. All reproduction is strictly prohibited.

7.2 Non-inclusion criteria

- Anticipated inability to follow up at 3 month
- Other obvious cause than PE for chest pain, syncope or dyspnea
- High clinical probability of PE (estimated by the physician gestalt as > 50%) or low clinical probability and PERC negative patients
- Low clinical probability (estimated by the physician gestalt as < 15%) and no item of the PERC score (*heart rate > 100, Sa02 < 95, unilateral leg swelling, hemoptysis, past history of thrombo-embolism, exogen estrogen intake, recent trauma or surgery, age ≥ 50)*
- Acute severe presentation (clinical signs of respiratory distress, hypotension, SpO2<90%, shock)
- Concurrent anticoagulation treatment
- Current diagnosed thrombo-embolic event (in the past 6 months)
- Prisoners
- Pregnancy
- No social security
- Participation in another intervention trial

7.3 Enrolment procedure

In participating centers, patients with suspicion of PE represent a high volume of ED visit. Previous retrospective studies [6,26,42] in these centers confirm that the potential for recruitment exceeds 30 eligible patients per month, from whom roughly 75% have a non-high gestalt clinical probability of PE [33]. Moreover, previous prospective studies with similar inclusion criteria achieved comparable recruitment target.
In particular, 08 of these 16 centers participated in the previous PROPER trial with similar design, and more restrictive inclusion criteria. Despite this, these 08 centers achieved the recruitment of 1420 patients in 10 months.

	**Number of participants**
Total number of participants to be included	1400
Number of sites	20
Enrolment period (months)	8
Number of participants/site	70
**Number of participants/site/month**	**8,75**

This document is the property of DRCI / AP-HP. All reproduction is strictly prohibited.

Expected number of patients recruited in the participating centers is as follows:

Name	First name	City	Center	Expected Recruitment / month	Total expected
Freund	Yonathan	Paris	Pitie (AP-HP)	12	96
Raynal	Pierre-Alexis	Paris	Saint Antoine (AP-HP)	10	80
Adnet	Frédérci	Paris	Avicenne	6	48
Chauvin	Anthony	Paris	Lariboisiere (AP-HP)	8	64
Khellaf	Medhi	Paris	Mondor (AP-HP)	6	48
Curac	Sonja	Paris	Beaujon	6	48
Fémy	Florent	Paris	HEGP (AP-HP)	8	64
Ganansia	Olivier	Paris	St Joseph	8	64
Choquet	Christophe	Paris	Bichat (AP-HP)	6	48
Guenezan	Jeremy	Poitiers	CHU Poitiers	8	64
Claret	Pierre-Geraud	Nimes	CHU Nimes	8	64
Goulet	Hélène	Paris	Tenon(AP-HP)	6	48
Tazarourte	Karim	Lyon	HC de Lyon	7	56
Le Borgne	Pierrick	Strasbourg	CHU Strasbourg	6	48
Montassier	Emmanuel	Nantes	CHU Nantes	8	64
Chouhied	Tahar	Nancy	CHU Nancy	8	64
Occelli	Céline	Nice	CHU Nice	8	64
Michelet	Pierre	Marseille	CHU Marseille	8	64
Wargon	Mathias	St Denis	CH Delafontaine	6	48
Laribi	Said	Tours	CHU Tours	8	64
Castro Arias	Lorena	Madrid	Hospital 12 de Octubre	6	48
Polo Lozano	Laura	Barcelona	Hospital de Sant Pau	6	48
Ruiz Artacho	Pedro	Madrid	Clínica Universitaria de Navarra	6	48
Jiménez	Sònia	Barcelona	Hospital Clinic	6	48
TOTAL				175	1400

8 TERMINATION AND EXIT RULES

There are a number of possible situations:

- Temporary suspension: the investigator must document the reason for suspending and resuming the treatment in the participant's source file and the case report form (CRF)
- Premature termination, but the participant remains enrolled in the study until the end of his/her participation: the investigator must document the reason

8.1 Criteria and procedure for premature withdrawals and exits from the study

- Participants may exit the study at any time and for any reason.

This document is the property of DRCI / AP-HP. All reproduction is strictly prohibited.

- The investigator can temporarily or permanently withdraw a participant from the study for any safety reason or if it is in the participant's best interests.
- If a participant leaves the study prematurely or withdraws consent, any data collected prior to the date of premature discontinuation may still be used.

- The case report form must list the various reasons why the participant exited or was withdrawn from the study:
  - ☐ Adverse reaction
  - ☐ Another medical issue
  - ☐ Personal reasons of the participant
  - ☐ Explicit withdrawal of consent

If a subject exits the trial, this will, in no way, affect the standard care received for his/her condition.

8.1.1 Procedure for replacing participants

Subjects who exit the study will not be replaced.

8.1.2 Full or partial cancellation of the study

AP-HP, as the sponsor, reserves the right to permanently suspend enrolment at any time if the enrolment targets have not been met.

9 EFFICACY ASSESSMENT

9.1 Description of parameters for assessing efficacy endpoints

Diagnosis of thrombo-embolic event will be made using usual criteria [43]:
For DVT: on the basis of an abnormal result on proximal compression ultrasonography
For PE: on the basis of a CTPA or angiography showing intraluminal defect, or a Ventilation/Perfusion lung scan showing a high-probability pattern.

9.2 Anticipated methods and timetable for measuring, collecting and analyzing the efficacy data

Recruited patients will be followed up at 3 months by phone interview or mail or email, or hospital visit in case they are still hospitalised. The primary endpoint is the occurrence of a thrombo-embolic event that has not been diagnosed in the ED at the inclusion visit (DVT or PE). A structured questionnaire will assess this eventuality of this occurrence. Patients will be asked whether they have had another visit to the hospital or physician appointment, and whether they had diagnostic tests for thrombo-embolic event (namely lower limbs Doppler ultrasound, CTPA or V/Q scan). They will also be asked whether an anticoagulation treatment was introduced during the follow up period.

- All suspected events will be collected, and their complete medical files will be sent for external adjudication of the primary endpoint by an adjudication committee of three independents experts, blinded to each other and blinded to the period allocation of the patient. All files with evidence of thrombo-embolic event and death with no evident underlying acute cause and will be sent to the adjudication committee for expertise

If the patient cannot be contacted, a next of kin or family member will be contacted. If not possible, the family physician will be contacted. If follow up is impossible, the investigators will contact the city hall and administrative service of his hometown to seek for possible death.

This reference methodology for outcome adjudication in PE studies has been used and described in all major diagnostic studies on PE [12,26,42,43].

10 SAFETY

This document is the property of DRCI / AP-HP. All reproduction is strictly prohibited.

During this research, adverse events (serious and non-serious) do not need to be reported to the sponsor. The report must instead be made as part of the vigilance procedure applicable to the intervention under investigation.

11 SPECIFIC COMMITTEES FOR THE STUDY

11.1 Steering Committee

Members of the committee: Pr Yonathan FREUND, Alexandra ROUSSEAU, Dr Anne-Laure Philippon, Pr Tabassome SIMON; (Paris, France).
Missions: design the study, define target population, define primary and secondary assessment criteria, monitor inclusion rate and follow up of the patients.

11.2 Endpoint Adjudication Committee

Members of the committee: Pr Andrea Penaloza (Bruxelles, Belgium), Pr Frédéric Lapostolle (Paris, France) and Pr Yann-Erick Claessens (Monaco, MC).

Missions: independently adjudicate the occurrence of likely thrombo-embolic event after 3 month follow up, in case of undocumented suspicion, or death.
Operating methods: for all patients that had an event during the 3 months follow up that could be related to a thrombo-embolic event, the medical record will be anonymized and blinded to the study period, and sent for external adjudication to the Endpoint Adjudication Committee.

12 DATA MANAGEMENT

12.1 Data collection

Data will be collected in an electronic case report form (e-CRF), devised by the study coordinator in collaboration with URC-EST. Data will be completed by the investigators for each visit of follow up with the help of a Clinical Research Technician (CRT) of URC-Est for AP-HP centers and of each center for others centers.

After an eligible patient is screened and fulfil all inclusion criteria, and no non-inclusion ones, and does not oppose to participating in the study. When included, the physician in charge or the local investigator will record the following data on an eCRF.
A local research assistant can help the physician in charge to this task, either the same day, (or retrospectively the following days if some data were not recorded) under the control of the local investigator or the treating physician. He will then complete and record all mandatory data in an electronic CRF.

Outcome data recorded at follow up will be entered in the same eCRF, as any serious adverse events that might occur.

12.2 Identification of data recorded directly in the CRF which will be considered as source data

For each recruited patient, besides usual clinical and biological data, we will collect the following specific items:
- Both the Revised Geneva Score and Wells score at presentation (including the 3 components of the YEARS rule).
- Any return visit to the hospital or to a physician during the follow up.
- All imaging studies that the patient has undergone during the follow up.
- Intake of anticoagulant regimen.

This document is the property of DRCI / AP-HP. All reproduction is strictly prohibited.

12.3 Right to access source data and documents

12.3.1 Data access

In accordance with GCP:
- the sponsor is responsible for ensuring all parties involved in the study agree to guarantee direct access to all locations where the study will be carried out, the source data, the source documents and the reports, for the purposes of the sponsor's quality control and audit procedures.
- the investigators will ensure the persons in charge of monitoring and auditing the research and of quality control have access to the documents and personal data strictly necessary for these tasks, in accordance with the statutory and regulatory provisions in force.

12.3.2 Source documents

The source documents are any original document or item that proves the existence or accuracy of a data-point or fact recorded during the study. Source documents will be kept by the investigator, or by the hospital in the case of hospital medical records, for 15 years.

12.3.3 Data protection

The persons responsible for the quality control of clinical studies (Article L.1121-3 of the Code de la Santé Publique - CSP (French Public Health Code) will take all necessary precautions to ensure the confidentiality of information relating to the research, the participants and in particular their identity and the results obtained.
These persons, as well as the investigators themselves, are bound by professional secrecy (in accordance with the conditions set out in Articles 226-13 and 226-14 of the French Criminal Code).
During and after the research, all data collected about the participants and sent to the sponsor by the investigators (or any other specialized collaborators) will be anonymized.
Under no circumstances will the names and addresses of the participants be shown.
The sponsor will ensure that each participant in the study has not opposed access to personal information about him/her strictly necessary for the purposes of quality control of the study.

12.4 Data processing and storage of research documents and data

12.4.1 Identification of the person responsible and the location for data processing

Data management will be performed by a data manager from URC-Est under the responsibility of Pr T. Simon. Statistical analysis will be performed by a biostatistician from URC-Est under the responsibility of Pr T. Simon.

12.4.2 Data entry

Data will be entered electronically via a web browser.

12.4.3 Procedures relating to data protection regulations

This study meets the definition in paragraph 2° of Article L.1121-1 (Law 2012-300 of 05/03/2012).According to Article L1122-1-4 of the Public Health Code: "In case of research involving the human person mentioned in 2 ° of Article L. 1121-1 whose methodological requirements are not compatible with the collection of consent under the conditions provided for in the second paragraph of the Article L. 1122-1-1, the protocol presented to the opinion of the committee for the protection of the persons concerned may provide that such consent is not sought and that the information provided for in Article L. 1122-1 is collective."

Declaration of compliance with the MR-001 "Reference Method"

This research is governed by the CNIL (French Data Protection Agency) “Reference Methodology for processing personal data used within the scope of health research” (amended MR-001). AP-HP, as sponsor of the research, has signed a declaration of compliance with this “Reference Methodology” Adapt based on the internal procedures of the entity managing the data.

This document is the property of DRCI / AP-HP. All reproduction is strictly prohibited.

The computer file used for this research is implemented in accordance with French (amended "Informatique et Libertés" law governing data protection) and European (General Data Protection Regulation – GDPR) regulations.

12.4.4 Archiving

All specific documents for Minimal Risk and Restriction research studies are to be archived by the investigator and the sponsor for 15 years after the end of the research.

This indexed archiving applies to:

- "Study" binders for the Investigator and the sponsor, containing (non-exhaustive list):
  - all successive versions of the protocol (identified by version no. and date), and its appendices
  - decisions of the CPP
  - correspondence
  - the enrolment list or register
  - the appendices specific to the research
  - Final report
  - The data collection documents

12.5 Ownership of the data

AP-HP is the owner of the data. The data cannot be used or disclosed to a third party without its prior permission.

13 STATISTICAL ASPECTS

13.1 Description of statistical methods to be used including the timetable for the planned interim analyses

No interim analysis is planned. Analysis will be performed at the end of the study after data review and freezing of data base.
Analyses will be performed using SAS® software (version 9.3 or updated version).
Baseline patient's characteristics will be considered at both with the cluster (center) and patient level. For the center level, characteristics at the beginning of the study will be described (there are no expected change between the two periods for cluster characteristics). Baseline characteristics of patients will be described globally and according to the group of intervention.
Continuous variables will be summarized using descriptive statistics, i.e number of subjects, mean, median, standard deviation (s.d), inter quartile range, minimum and maximum. Categorical variables will be summarized by frequency and percentage.

Principal criteria analysis:

Since this is a non-inferiority study, analysis of the principal criterion will be performed on per protocol population. A sensitivity analysis will be performed on ITT population.
Thrombo-embolic event (TE event) will be defined by: DVT (assessed by proximal compression ultrasonography) or PE (a CTPA or angiography showing intraluminal defect, or a Ventilation/Perfusion lung scan showing a high-probability pattern). A generalized linear regression mixed model with Poisson distribution (log link) will be performed, taking into account a random effect for each center and considering period and strategy-by-period interaction as fixed effects. The decision rule will be based on the upper bound of the 95% two sided confidence interval of the relative risk of TE events.

In a second time, sensitivity analysis will be performed using the 95% two sided confidence interval of the difference of percentage of TE events between groups will be performed. If the upper bound of the confidence interval is above the 1.35% of difference, the inferiority hypothesis of the intervention group will be rejected.

This document is the property of DRCI / AP-HP. All reproduction is strictly prohibited.

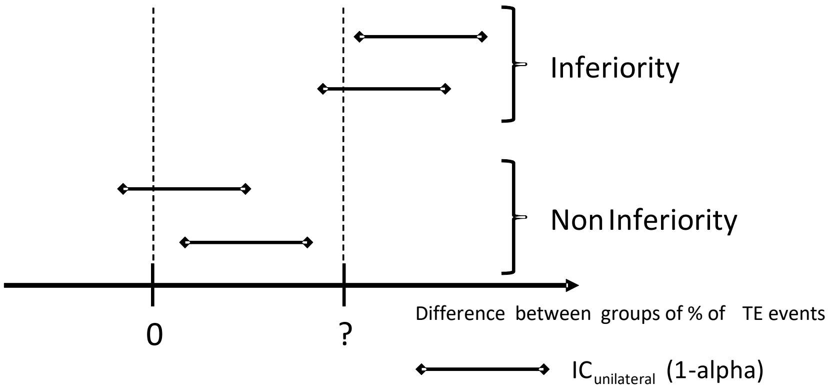

Secondary evaluation criteria:

Secondary criteria will be compared between groups on the ITT population and under superiority hypothesis.
Proportion of irradiative imaging, of undue onset of anticoagulation regimen, of hospital admission following the ED visit, of all causes hospital readmission at 3 month and of deaths from all causes at 3 months will be compared between groups by using a
generalized linear regression mixed models with Poisson distribution (log link). A random effect for each cluster will be considered and considered fixed effects will be period and strategy-by-period interaction If the number of events is sufficient, generalized linear regression mixed model with Bernoulli distribution (logit link) will be performed. Proportion difference between groups and its 95% confidence interval will be calculated.

The ED length of stay will be compared between the two periods by using a linear regression mixed model A random effect for each cluster will be considered and considered fixed effects will be period and strategy-by-period interaction. In case of non-normality distribution of the interest variable, a transformation could be performed or a model appropriate to data distribution could be selected.

Difference between groups of proportion of patients with diagnosed pulmonary embolism at 3 month follow-up excluding the isolated sub-segmental pulmonary embolism, among patients in whom PE has been initially ruled out, will be calculated as well its 95% confidence interval.
If possible, generalized linear regression mixed models with Poisson distribution (log link) will be performed. A random effect for each cluster will be considered and considered fixed effects will be: period and strategy-by-period interaction If the number of events is sufficient, generalized linear regression mixed model with Bernoulli distribution (logit link) will be performed.

Proportion of patients with diagnosed thrombo-embolic event at 3 month follow-up (either a PE or a deep venous thrombosis), among patients in whom PE has been initially ruled out by the PEPS score will be described with its 95% confidence interval.

Sensitivity analysis will be performed on the per protocol population.

13.2 Calculation hypotheses for the number of subjects required and the result

In the recent recommendations from International Society of Thrombosis and Hemostasis on studies for PE diagnosis in the ED, it has been suggested that the **maximal acceptable failure rate of a tested strategy should not exceed 1.85% (Dronkers JTH 2017).[29]**
The recent large European prospective cohort studies on PE diagnosis (PROPER, PERCEPIC, YEARS, ADJUST-PE) reported a failure rate of 0.1-0.5%.
Sample size under non inferiority hypothesis:
With an anticipated failure rate of **0.5% in the control group, a non-inferiority margin set at 1.35%** (according to the ISTH recommendations so the upper bound of the 95% CI of the failure

This document is the property of DRCI / AP-HP. All reproduction is strictly prohibited.

rate in the intervention group will not exceed 1.85%)beta=20% and one-sided alpha=2.5%, N1=857 patients are needed (East 6, Cytel).
Cluster design effect hypothesis:

- 20 clusters and 2 periods.
- Intraclass correlation coefficient (CCIC) = 0.018
- Inter period correlation (η) = 0.0115
- Mean cluster size for one period (m) = 22 patients
- Cluster design effect: D= (1+(m-1)xCCIC) - η = 1.37.

Sample size needed = D x N1 = 1174 patients.

Accounting for up to 5% of non-evaluable patients, we need to recruit 1234 patients.

Beside the non-inferiority analysis, it is of utmost importance that the upper bound of the 95%CI of the failure rate of the tested modified diagnostic strategy is below 1.85%, whatever the rate of the control group is.
Our retrospective study reported an anticipated failure rate at 0.85% but focused only on low risk patients. Therefore, **with an anticipated failure rate of 1% in the intervention period, the sample size of this group should be of at least 700 to respect the maximal upper bound of 1.85%.**
Therefore, for a **conservative** approach of these 2 conditions (non-inferiority margin at 1.35% and maximal upper bound of the 95%CI below 1.85%), **1400 (700 in each group) subjects will be needed in this study..**

13.3 Anticipated level of statistical significance

All tests will be performed at 5%.

13.4 Statistical criteria for termination of the study

Not applicable.

13.5 Method for taking into account missing, unused or invalid data

Missing value for the principal criteria will be considered as failure, whatever the period considered. Others missing data will not be replaced.

13.6 Management of modifications made to the analysis plan for the initial strategy

Modification made in analysis will be documented in the final report.

13.7 Choice of individuals to include in the analyses

ITT population: all included patients according to the period assigned by the randomization to the center, regardless of the strategy effectively received by the patient.

Per protocol population: all included patients without major protocol deviation that would be:

- No respect of selection criteria,
- No respect of strategy assigned by randomization (cross-over for example),
- Missing value for the principal criteria,
- Other major protocol deviation identified during data review and freezing of data base.

13.8 Medico-economic evaluation

This first economic evaluation of the innovative diagnostic strategy of PE follows the recommendations from the French national health authority and the CHEERS statement for single-trial based studies
(http://www.has-sante.fr/portail/upload/docs/application/pdf/201111/guide_methodo_vf.pdf

79. Husereau D, Drummond M, Petrou S, et al. Consolidated Health Economic Evaluation Reporting Standards (CHEERS) statement. BMJ 2013;346:f1049.).

This document is the property of DRCI / AP-HP. All reproduction is strictly prohibited.

The economic evaluation has to consider 3 possibilities: cost minimization which would assume the equivalence of strategies on the effectiveness endpoint; incremental cost effectiveness /decremental cost effectiveness; or dominance. Given the non-inferiority hypothesis the primary analysis is a cost-minimization analysis prolonged by a cost-effectiveness sensitivity analysis on the cost per major event (death and TE event averted). (Briggs AH, O'Brien BJ. The death of cost-minimization analysis? Health Econ. 2001;10:179-84.). The perspective of the analysis is the healthcare system and the time horizon 3 months. The cost minimization/cost effectiveness analysis will use the difference in the safety of each strategy as the effectiveness criterion in accordance with previous studies (Sterne JA, Bodalia PN, Bryden PA, Davies PA, López-López JA, Okoli GN, Thom HH, Caldwell DM, Dias S, Eaton D, Higgins JP, Hollingworth W, Salisbury C, Savović J, Sofat R, Stephens-Boal A, Welton NJ, Hingorani AD.Oral anticoagulants for primary prevention, treatment and secondary prevention of venous thromboembolic disease, and for prevention of stroke in atrial fibrillation: systematic review, network meta-analysis and cost-effectiveness analysis.Health Technol Assess. 2017 Mar;21(9):1-386).

The economic evaluation is based on the entire population of patients included in the trial.

Estimating resources and costs

This is a single study-based economic evaluation: resource utilization will be collected prospectively at the patient level using the study case report form supplemented by data from the hospital claims database for the entire duration of the study period.

Medical care costs for the index admission and in-trial follow-up period are assessed using a combination of resource-based and event-based methods. In-hospital resource utilization will be described based on diagnosis and procedural codes and length of stay for the entire duration of the index admission and subsequent hospital stays. The total length of stay and discharge information (DRG) will be extracted from the hospital information system for the index admission and event-related subsequent admissions during the 1 month follow up period. Length of stay in the ICU, step down units and conventional wards will be retrieved. In France, hospital costs will be assigned based on the Severity-Diagnosis Related Groups. Specific ICU costs are assigned based upon the total duration of ICU stay. The cost of each admission will be estimated from the national cost study, using the actual length of stay and the per diem cost stratified by DRG.
We will use the latest mean cost available (or tariff if costs are not available).

In Spain, some hospitals experiment DRGs and produce DRG-specific costs. These costs cannot be extrapolated at the national level. Other hospitals calculate cost per department (eg cardiology) per day, aggregated at the regional level for budgeting purposes. We will record length of stay for all cardiac-related admissions and DRGs in hospitals where the information is available and derive average daily hospital costs (regional) to be used in cost comparisons.

Out of hospital resources will be estimated from the CRF and patients interviews during the follow up visits (35 days and 90 days). We will collect information on consultations, medications, laboratory tests. Out of hospital resources will be valued using the latest price/ tariff schedule.
Missing cost data: it is expected that all in-hospital resources will be collected given the fact that a hospital admission is a serious adverse event.
Total costs under current diagnostic and treatment protocols will be estimated for each patient for the index attendance/admission and over 3 months of follow-up.

Discount rate

In view of the short duration of this study, costs and benefits are not discounted.

Cost effectiveness study

The primary outcome measure for the economic evaluation is 3-month cumulated adverse events defined as death or TE.

This document is the property of DRCI / AP-HP. All reproduction is strictly prohibited.

Data analysis

The unit of analysis is the patient. Costs will be presented as means with 2.5 to 97.5% bootstrapped intervals. Between-group comparisons of costs will be performed using the bootstrap t-test. A joint comparison of costs and effects will be performed by nonparametric bootstrapping with 1,000 resamples and the probabilities of better/worse performance of modified diagnostic algorithm and higher/lower costs will be determined.

14 QUALITY CONTROL AND ASSURANCE

14.1 General organization

The sponsor must ensure the safety and respect of individuals who didn't oppose to participate in the study. The sponsor must have a quality assurance system for monitoring the implementation of the study at the research centers.
The sponsor will establish a system for opening the research centers and may also implement a data quality control system.

14.1.1 Strategy for site opening

The strategy for opening the centers will be determined before the research begins.

14.1.2 Data quality control

A Clinical Research Associate (CRA) appointed by the sponsor will be responsible for the proper running of the study, for collecting, documenting, recording and reporting all handwritten data, in accordance with the Standard Operating Procedures applied within the DRCI.
The investigator and the members of the investigator's team agree to make themselves available during regular Quality Control visits by the Clinical Research Associate.

14.2 Case report forms

All information required by the protocol must be entered in the case report forms. The data must be collected as and when they are obtained, and must be written clearly and legibly. Any missing data must be coded.
Every site will have access to the electronic case report forms via a web-based data collection system. Investigators will be given instructions for using this tool.
Using on-line case report forms means the CRA can view the data quickly and remotely. The investigator is responsible for the accuracy, quality and relevance of all the data. In addition, there are consistency checks to ensure the data are verified immediately upon being entered. The investigator must validate any changes to the values in the case report form. An audit trail will be kept of all changes. A justification can be added when applicable, as a comment.
A print-out, authenticated (signed and dated) by the investigator, will be requested at the end of the research. The sponsor will keep the original. The investigator must keep a copy.

14.3 Management of non-compliances

Any events that occur as a result the investigator or any other individual involved in conducting the study failing to comply with the protocol, standard operating procedures or statutory and regulatory requirements must be recorded in a declaration of non-compliance and sent to the sponsor.

The sponsor has its own procedures for managing these non-compliances.

14.4 Audits

The investigators agree to accept the quality assurance audits carried out by the sponsor as well as the inspections carried out by the competent authorities. All data, documents and reports may be subject to regulatory audits. These audits and inspections cannot be refused on the grounds of medical secrecy.

This document is the property of DRCI / AP-HP. All reproduction is strictly prohibited.

An audit can be carried out at any time by independent individuals appointed by the sponsor. The aim of the audits is to ensure the quality of the study, the validity of the results and compliance with the legislation and regulations in force.

The persons who manage and monitor the study agree to comply with the sponsor's audit requirements.
The audit may encompass all stages of the study, from the development of the protocol to the publication of the results and the storage of the data used or produced as part of the study.

14.5 Principal Investigator's declaration of responsibility

Before starting the study, each investigator will give the sponsor's representative a signed and dated copy of his/her most recent curriculum vitæ, produced within the past year, and RPPS number (Répertoire Partagé des Professionnels de Santé, Collective Database of Health Professionals). The CV must describe any previous participation in clinical research and related training.

Each investigator will agree to comply with legislation and to conduct the study in line with regulations, in accordance with the Declaration of Helsinki.

The Principal Investigator at each participating site will sign a declaration of responsibility (standard DRCI document) which will be sent to the sponsor's representative.
The investigators and their co-workers will sign a delegation form specifying each person's role and must supply their CV.

15 ETHICAL AND LEGAL CONSIDERATIONS

15.1 Methods for informing the research participants

All ED eligible patients will be informed of the study by an information form in participating centers. The treating physician or the local investigator will explain the rationale and objectives of the study. An information note will be given to him, and he will be able to discuss the study with the physician or local investigator or research assistant. If the patient does express his freely-given oral consent, he can be included in the study.
In addition, the investigator will specify in the person's medical file the person's participation in the research, the procedures for obtaining his/her oral consent.

15.2 Prohibition of concomitant clinical studies participation and exclusion period after the study

During his participation to the MODIGLIA-NI study (3 months), the subject may not participate in other interventional studies.
There is no exclusion period after the study participation.

15.3 Compensation for participants

No compensation is anticipated for the patients as compensation for the inconveniences relating to the research.

15.4 Legal obligations

Assistance Publique Hôpitaux de Paris (AP-HP) is the sponsor of this study and has delegated powers to its Clinical Research and Innovation Department (DRCI) in order to conduct the study in accordance with Article L.1121-1 of the Code de la Santé Publique - CSP (French Public Health Code).AP-HP reserves the right to terminate the study at any time for medical or administrative reasons. In this case, the investigator will be informed accordingly.

This document is the property of DRCI / AP-HP. All reproduction is strictly prohibited.

15.5 Request for approval from the CPP

AP-HP, as sponsor, obtains prior approval from the CPP for its Minimal Risks and burden research studies, within the scope of the Board's authority and in accordance with statutory and regulatory requirements.

15.6 Informing the ANSM

The AP-HP will send the approval from the CPP and the summary of the protocol to the ANSM, for information.

15.7 Declaration of compliance with the MR-003 "Reference Method"

AP-HP, the study sponsor, has signed a declaration of compliance with this "Reference Method".

15.8 Modifications to the study

Any substantial amendment made to the protocol must be sent to the sponsor for approval. Once approval has been received from the sponsor, it must also obtain approval from the CPP before the amendment can be implemented.
The information form can be revised if necessary, in particular if there is substantial amendment to the study or if adverse reactions occur.

16 FUNDING AND INSURANCE

16.1 Sources of monetary support

Ministry of health: Programme Hospitalier de Recherche Clinique 2018 (PHCR 2018).

16.2 Insurance

For the duration of the study, the Sponsor will take out an insurance policy covering the sponsor’s own third party liability as well as the third party liability of all the doctors involved in the study. The sponsor will also provide full compensation for any damages caused by the study to the study participants and their beneficiaries, unless the sponsor can prove that the harm is not the fault of the sponsor or any agent. Compensation cannot be refused on the grounds of a third party act or the voluntary withdrawal of the person who initially didn't oppose to participate in the study.

Assistance Publique-Hôpitaux de Paris (AP-HP) has taken out insurance with HDI-GLOBAL SE through BIOMEDIC-INSURE (contract no. 0100518814033 190054), covering its own third party liability and that of any agent (doctor or research staff), in accordance with Article L.1121-10 of the Code de la Santé Publique - CSP (French Public Health Code).

This document is the property of DRCI / AP-HP. All reproduction is strictly prohibited.

17 PUBLICATION RULES

17.1 Mention of AP-HP affiliation for projects sponsored by AP-HP

If an author has several affiliations, the order in which the institutions are mentioned (AP-HP, University, INSERM, etc.) is not important.

17.2 Mention of the sponsor AP-HP (DRCI) in the acknowledgements of the text

"The sponsor was Assistance Publique – Hôpitaux de Paris (Clinical Research and Innovation Department)".

17.3 Mention of the funder in the acknowledgements of the text

Acknowledgement will include: "The study was funded by a grant from Programme Hospitalier de Recherche Clinique - PHRC 2018 (Ministry of Health)".
This research program will be registered on the website http://clinicaltrials.gov/ (include the registration number once registered).

17.4 Author lists

The authorship will be as follows:
Freund Y, Philippon AL, Jiménez S, Inv 1, Inv 2, … inv 15, Durand Zaleski I, Cachanado M, Simon T.
Inv N being the investigator of the N centers with the highest recruitement.

18 REFERENCES

1. Oger E. Incidence of venous thromboembolism: a community-based study in Western France. EPI-GETBP Study Group. Groupe d'Etude de la Thrombose de Bretagne Occidentale. *Thromb Haemost*. 2000;83(5):657-660.

2. Cohen AT, Agnelli G, Anderson FA, et al. Venous thromboembolism (VTE) in Europe. The number of VTE events and associated morbidity and mortality. *Thromb Haemost*. 2007;98(4):756-764.

3. Goldhaber SZ, Visani L, De Rosa M. Acute pulmonary embolism: clinical outcomes in the International Cooperative Pulmonary Embolism Registry (ICOPER). *Lancet*. 1999;353(9162):1386-1389.

4. American College of Emergency Physicians Clinical Policies Committee, Clinical Policies Committee Subcommittee on Suspected Pulmonary Embolism. Clinical policy: critical issues in the evaluation and management of adult patients presenting with suspected pulmonary embolism. *Ann Emerg Med*. 2003;41(2):257-270. doi:10.1067/mem.2003.40

5. Konstantinides SV, Torbicki A, Agnelli G, et al. 2014 ESC guidelines on the diagnosis and management of acute pulmonary embolism. *Eur Heart J*. 2014;35(43):3033-3069, 3069a-3069k. doi:10.1093/eurheartj/ehu283

6. Le Gal G, Righini M, Roy P-M, et al. Prediction of pulmonary embolism in the emergency department: the revised Geneva score. *Ann Intern Med*. 2006;144(3):165-171.

7. Wells PS, Anderson DR, Rodger M, et al. Derivation of a simple clinical model to categorize patients probability of pulmonary embolism: increasing the models utility with the SimpliRED D-dimer. *Thromb Haemost*. 2000;83(3):416-420.

This document is the property of DRCI / AP-HP. All reproduction is strictly prohibited.

8. Lucassen W, Geersing G-J, Erkens PMG, et al. Clinical decision rules for excluding pulmonary embolism: a meta-analysis. *Ann Intern Med*. 2011;155(7):448-460. doi:10.7326/0003-4819-155-7-201110040-00007

9. Penaloza A, Verschuren F, Meyer G, et al. Comparison of the unstructured clinician gestalt, the wells score, and the revised Geneva score to estimate pretest probability for suspected pulmonary embolism. *Ann Emerg Med*. 2013;62(2):117-124.e2. doi:10.1016/j.annemergmed.2012.11.002

10. Chunilal SD, Eikelboom JW, Attia J, et al. DOes this patient have pulmonary embolism? *JAMA*. 2003;290(21):2849-2858. doi:10.1001/jama.290.21.2849

11. Roy P-M, Bichri A, Bouet R, Mottier H. Embolie pulmonaire : algorithmes diagnostiques. http://www.sfar.org/acta/dossier/archives/mu07/html/mu07_07/urg07_07.htm. Published 2007. Accessed December 23, 2013.

12. Perrier A, Roy P-M, Aujesky D, et al. Diagnosing pulmonary embolism in outpatients with clinical assessment, D-dimer measurement, venous ultrasound, and helical computed tomography: a multicenter management study. *Am J Med*. 2004;116(5):291-299. doi:10.1016/j.amjmed.2003.09.041

13. Righini M, Nendaz M, Le Gal G, Bounameaux H, Perrier A. Influence of age on the cost-effectiveness of diagnostic strategies for suspected pulmonary embolism. *J Thromb Haemost JTH*. 2007;5(9):1869-1877. doi:10.1111/j.1538-7836.2007.02667.x

14. Le Gal G, Bounameaux H. Diagnosing pulmonary embolism: running after the decreasing prevalence of cases among suspected patients. *J Thromb Haemost JTH*. 2004;2(8):1244-1246. doi:10.1111/j.1538-7836.2004.00795.x

15. Kline JA, Mitchell AM, Kabrhel C, Richman PB, Courtney DM. Clinical criteria to prevent unnecessary diagnostic testing in emergency department patients with suspected pulmonary embolism. *J Thromb Haemost JTH*. 2004;2(8):1247-1255. doi:10.1111/j.1538-7836.2004.00790.x

16. van der Hulle T, Cheung WY, Kooij S, et al. Simplified diagnostic management of suspected pulmonary embolism (the YEARS study): a prospective, multicentre, cohort study. *Lancet Lond Engl*. 2017;390(10091):289-297. doi:10.1016/S0140-6736(17)30885-1

17. Singh B, Parsaik AK, Agarwal D, Surana A, Mascarenhas SS, Chandra S. Diagnostic accuracy of pulmonary embolism rule-out criteria: a systematic review and meta-analysis. *Ann Emerg Med*. 2012;59(6):517-520.e1-4. doi:10.1016/j.annemergmed.2011.10.022

18. Pernod G, Caterino J, Maignan M, et al. D-Dimer Use and Pulmonary Embolism Diagnosis in Emergency Units: Why Is There Such a Difference in Pulmonary Embolism Prevalence between the United States of America and Countries Outside USA? *PloS One*. 2017;12(1):e0169268. doi:10.1371/journal.pone.0169268

19. Penaloza A, Soulié C, Moumneh T, et al. Pulmonary embolism rule-out criteria (PERC) rule in European patients with low implicit clinical probability (PERCEPIC): a multicentre, prospective, observational study. *Lancet Haematol*. 2017;4(12):e615-e621. doi:10.1016/S2352-3026(17)30210-7

This document is the property of DRCI / AP-HP. All reproduction is strictly prohibited.

20. Wiener RS, Schwartz LM, Woloshin S. When a test is too good: how CT pulmonary angiograms find pulmonary emboli that do not need to be found. *BMJ*. 2013;347(jul02 2):f3368-f3368. doi:10.1136/bmj.f3368

21. Wiener RS, Schwartz LM, Woloshin S. Time Trends in Pulmonary Embolism in the United States: Evidence of Overdiagnosis. *Arch Intern Med*. 2011;171(9):831-837. doi:10.1001/archinternmed.2011.178

22. Schissler AJ, Rozenshtein A, Kulon ME, et al. CT Pulmonary Angiography: Increasingly Diagnosing Less Severe Pulmonary Emboli. *PLoS ONE*. 2013;8(6):e65669. doi:10.1371/journal.pone.0065669

23. Mitchell AM, Kline JA. Contrast nephropathy following computed tomography angiography of the chest for pulmonary embolism in the emergency department. *J Thromb Haemost JTH*. 2007;5(1):50-54. doi:10.1111/j.1538-7836.2006.02251.x

24. Einstein AJ, Henzlova MJ, Rajagopalan S. Estimating risk of cancer associated with radiation exposure from 64-slice computed tomography coronary angiography. *JAMA J Am Med Assoc*. 2007;298(3):317-323. doi:10.1001/jama.298.3.317

25. Cochran ST, Bomyea K, Sayre JW. Trends in adverse events after IV administration of contrast media. *AJR Am J Roentgenol*. 2001;176(6):1385-1388. doi:10.2214/ajr.176.6.1761385

26. Perrier A, Roy P-M, Sanchez O, et al. Multidetector-Row Computed Tomography in Suspected Pulmonary Embolism. *N Engl J Med*. 2005;352(17):1760-1768. doi:10.1056/NEJMoa042905

27. Roy P-M, Durieux P, Gillaizeau F, et al. A computerized handheld decision-support system to improve pulmonary embolism diagnosis: a randomized trial. *Ann Intern Med*. 2009;151(10):677-686. doi:10.7326/0003-4819-151-10-200911170-00003

28. Freund Y, Rousseau A, Guyot-Rousseau F, et al. PERC rule to exclude the diagnosis of pulmonary embolism in emergency low-risk patients: study protocol for the PROPER randomized controlled study. *Trials*. 2015;16:537. doi:10.1186/s13063-015-1049-7

29. Dronkers CEA, van der Hulle T, Le Gal G, et al. Towards a tailored diagnostic standard for future diagnostic studies in pulmonary embolism: communication from the SSC of the ISTH. *J Thromb Haemost*. 2017;15(5):1040-1043. doi:10.1111/jth.13654

30. Freund Y, Cachanado M, Aubry A, et al. Effect of the Pulmonary Embolism Rule-Out Criteria on Subsequent Thromboembolic Events Among Low-Risk Emergency Department Patients: The PROPER Randomized Clinical Trial. *JAMA*. 2018;319(6):559-566. doi:10.1001/jama.2017.21904

31. Kabrhel C, Van Hylckama Vlieg A, Muzikanski A, et al. Multicenter Evaluation of the YEARS Criteria in Emergency Department Patients Evaluated for Pulmonary Embolism. *Acad Emerg Med Off J Soc Acad Emerg Med*. March 2018. doi:10.1111/acem.13417

32. Gorlicki J, Penaloza A, Germeau B, et al. Safety of the combination of PERC and YEARS rules in patients with low clinical probability of PE: a retrospective analysis of two large European cohorts. *Acad Emerg Med Off J Soc Acad Emerg Med*. June 2018. doi:10.1111/acem.13508

This document is the property of DRCI / AP-HP. All reproduction is strictly prohibited.

33. Penaloza A, Verschuren F, Dambrine S, Zech F, Thys F, Roy P-M. Performance of the Pulmonary Embolism Rule-out Criteria (the PERC rule) combined with low clinical probability in high prevalence population. *Thromb Res*. 2012;129(5):e189-193. doi:10.1016/j.thromres.2012.02.016

34. Jiménez D, Yusen RD, Otero R, et al. Prognostic models for selecting patients with acute pulmonary embolism for initial outpatient therapy. *Chest*. 2007;132(1):24-30. doi:10.1378/chest.06-2921

35. Moores L, Aujesky D, Jiménez D, et al. Pulmonary Embolism Severity Index and troponin testing for the selection of low-risk patients with acute symptomatic pulmonary embolism. *J Thromb Haemost JTH*. 2010;8(3):517-522. doi:10.1111/j.1538-7836.2009.03725.x

36. Donzé J, Le Gal G, Fine MJ, et al. Prospective validation of the Pulmonary Embolism Severity Index. A clinical prognostic model for pulmonary embolism. *Thromb Haemost*. 2008;100(5):943-948.

37. Newman DH, Schriger DL. Rethinking testing for pulmonary embolism: less is more. *Ann Emerg Med*. 2011;57(6):622-627.e3. doi:10.1016/j.annemergmed.2011.04.014

38. Forero R, Hillman KM, McCarthy S, Fatovich DM, Joseph AP, Richardson DB. Access block and ED overcrowding. *Emerg Med Australas EMA*. 2010;22(2):119-135. doi:10.1111/j.1742-6723.2010.01270.x

39. Forero R, McCarthy S, Hillman K. Access block and emergency department overcrowding. *Crit Care*. 2011;15(2):216. doi:10.1186/cc9998

40. Guttmann A, Schull MJ, Vermeulen MJ, Stukel TA. Association between waiting times and short term mortality and hospital admission after departure from emergency department: population based cohort study from Ontario, Canada. *BMJ*. 2011;342:d2983.

41. Campbell MK, Elbourne DR, Altman DG. CONSORT statement: extension to cluster randomised trials. *BMJ*. 2004;328(7441):702-708.

42. Righini M, Le Gal G, Aujesky D, et al. Diagnosis of pulmonary embolism by multidetector CT alone or combined with venous ultrasonography of the leg: a randomised non-inferiority trial. *The Lancet*. 2008;371(9621):1343-1352. doi:10.1016/S0140-6736(08)60594-2

43. Righini M, Van Es J, Den Exter PL, et al. Age-adjusted D-dimer cutoff levels to rule out pulmonary embolism: the ADJUST-PE study. *JAMA J Am Med Assoc*. 2014;311(11):1117-1124. doi:10.1001/jama.2014.2135

This document is the property of DRCI / AP-HP. All reproduction is strictly prohibited.

19 ADDENDA

All addenda and the log of addenda versions are attached, separately to the protocol. All addenda can be modified (change of addendum version) without modifying the protocol version.

This document is the property of DRCI / AP-HP. All reproduction is strictly prohibited.

19.1 List of investigators

Centre n°	Etablissement	Service (Chef de service)	Investigateur principal (Titre - Nom - Prénom)	Adresse Postale du centre	Adresse Electronique
001	**Hôpital La Pitié-Salpêtrière - Paris**	Service des Urgences (Pr Bruno RIOU)	Dr Yonathan FREUND	83 Boulevard de l'hôpital 75013 Paris	yonatman@gmail.com
002	**Hôpital Avicenne - Bobigny**	Samu 93 (Pr Frédéric ADNET)	Pr Frédéric ADNET	125 Route de Stalingrad 93000 Bobigny	frederic.adnet@aphp.fr
003	**Hôpital Saint-Antoine - Paris**	Service d'Accueil des Urgences (Pr Dominique PATERON)	Dr Pierre-Alexis RAYNAL	184 Rue du faubourg Saint-Antoine 75012 Paris	raynalpa@gmail.com
004	**Hôpital Européen Georges Pompidou - Paris**	Service d'Accueil des Urgences (Pr Philippe JUVIN)	Dr Florent FEMY	20 Rue Leblanc 75015 Paris	florent.femy@gmail.com
005	**CHRU Hôpital Bretonneau - Tours**	Département de Médecine d'Urgences (Pr Said LARIBI)	Pr Said LARIBI	2 Boulevard Tonnellé 37000 Tours	s.laribi@chu-tours.fr
006	**CHRU Hôpital Central - Nancy**	SAMU 54 (Dr Lionel NACE)	Dr Tahar CHOUIHED	CHRU Nancy CO 60034 54035 Nancy Cedex	t.chouihed@chru-nancy.fr
007	**Hôpital Henri Mondor - Créteil**	Service des Urgences (Pr Mehdi KHELLAF)	Pr Mehdi KHELLAF	51 Avenue du Maréchal de Lattre de Tassigny 94010 Créteil	mehdi.khellaf@aphp.fr
008	**Hôpital Lariboisière - Paris**	Service des Urgences/SMUR Pr Patrick PLAISANCE	Dr Anthony CHAUVIN	2 Rue Ambroise Paré 75006 Paris	anthony.chauvin@aphp.fr
009	**Hôpital Saint-Joseph - Paris**	Service des Urgences et lits d'Urgence (Dr Olivier GANANSIA)	Dr Olivier GANANSIA	185 Rue Raymond Losserand 75014 Paris	oganansia@hpsj.fr
010	**CHU Hôpital Hôtel Dieu - Nantes**	Services des Urgences (Pr Eric BATARD)	Dr Emmanuel MONTASSIER	1 Place Antoine Ricordeau 44093 Nantes Cedex 1	emmanuel.montassier@chu-nantes.fr
011	**Hôpital Beaujon - Clichy**	Service des Urgences (Dr Sonja CURAC)	Dr Sonja CURAC	100, bd du Gal Leclerc 92110 Clichy	sonja.curac@aphp.fr
012	**Hôpital Bichat - Paris**	Service des Urgences (Dr Christophe CHOQUET)	Dr Christophe CHOQUET	46, rue Henri-Huchard 75018 PARIS	christophe.choquet@aphp.fr
013	**CHU de Poitiers**	Service des Urgences (Pr Olivier MIMOZ)	Dr Jérémy GUENEZAN	2 rue de la milétrie 86021 Poitiers	jeremyguenezan@gmail.com

This document is the property of DRCI / AP-HP. All reproduction is strictly prohibited.

**014**	**Hôpital Tenon - Paris**	Service des Urgences (Dr Hélène GOULET)	Dr Hélène GOULET	2, rue de la Chine 75020 Paris	helene.goulet@aphp.fr
**015**	**CHU de Strasbourg**	Service des Urgences (Pr Pascal BILBAULT)	Dr Pierrick LE BORGNE	1 Av Molière 67000 Strasbourg	pierrick.le-borgne@chru-strasbourg.fr
**016**	**Hôpital Delafontaine – Saint-Denis**	Service des Urgences	Dr Mathias WARGON	2 Rue du Dr Delafontaine, 93200 Saint-Denis	Mathias.wargon@ch-stdenis.fr
**017**	**Hospital 12 de octubre - Madrid**	Service des Urgences	Dr Lorena CASTRO ARIAS	Av. de Córdoba, s/n, 28041 Madrid Espagne	lcastroa@salud.madrid.org
**018**	**Hospital de Sant Pau - Barcelone**	Service de médecine	Dr Laura POLO LOZANO	Carrer de Sant Antoni Maria Claret, 167<br>08025 Barcelona Espagne	llozano@santpau.cat
**019**	**Clinica Universitaria de Navarra - Madrid**	Service de médecine	Dr Pedro RUIZ ARTACHO	Calle Marquesado de Sta. Marta, 1, 28027<br>Madrid Espagne	ruizpedroc@gmail.com
**020**	**Hospital Clinic - Barcelone**	Service des Urgences (Dr. Antoni Castells)	Dr Sonia JIMENEZ HERNANDEZ	Villarroel, 170<br>08036 Barcelona Espagne	SJIMENEZ@clinic.cat

This document is the property of DRCI / AP-HP. All reproduction is strictly prohibited.
