# Supplementary material for: MOdified DIagnostic strateGy to safely ruLe-out pulmonary embolism In the emergency depArtment: study protocol for the Non-Inferiority MODIGLIANI cluster cross-over randomized trial
Source: Trials. 2020 Jun 3;21:458. doi: 10.1186/s13063-020-04379-y (PMC7268276; doi:10.1186/s13063-020-04379-y)
Supplement: Supplementary file 2 — Additional file 2. Statistical analysis plan. [file 13063_2020_4379_MOESM2_ESM.pdf]

1 STATISTICAL ASPECTS

1.1 Description of statistical methods to be used including the timetable for the planned interim analyses

No interim analysis is planned. Analysis will be performed at the end of the study after data review and freezing of data base.
Analyses will be performed using SAS® software (version 9.3 or updated version).
Baseline patient's characteristics will be considered at both with the cluster (center) and patient level. For the center level, characteristics at the beginning of the study will be described (there are no expected change between the two periods for cluster characteristics). Baseline characteristics of patients will be described globally and according to the group of intervention.
Continuous variables will be summarized using descriptive statistics, i.e number of subjects, mean, median, standard deviation (s.d), inter quartile range, minimum and maximum. Qualitative variables will be summarized by frequency and percentage.

Principal criteria analysis:

Since this is a non-inferiority study, analysis of the principal criterion will be performed on per protocol population. A sensitivity analysis will be performed on ITT population.
Thrombo-embolic event (TE event) will be defined by: DVT (assessed by proximal compression ultrasonography) or PE (a CTPA or angiography showing intraluminal defect, or a Ventilation/Perfusion lung scan showing a high-probability pattern). Given the relatively rare occurrence of TE events, a Poisson model for proportions was chosen for the primary outcome analysis. Given the relatively rare occurrence of TE events,, a generalized linear regression mixed model with Poisson distribution (log link) will be performed (Poisson model for proportions), taking into account a random effect for each center and considering period and strategy-by-period interaction as fixed effects. The decision rule will be based on the upper bound of the 95% two sided confidence interval of the incidence rate ratio of TE events.

In a second time, sensitivity analysis will be performed using the 95% two sided confidence interval of the difference of percentage of TE events between groups will be performed. If the upper bound of the confidence interval is above the 1.35% of difference, the inferiority hypothesis of the intervention group will be rejected.

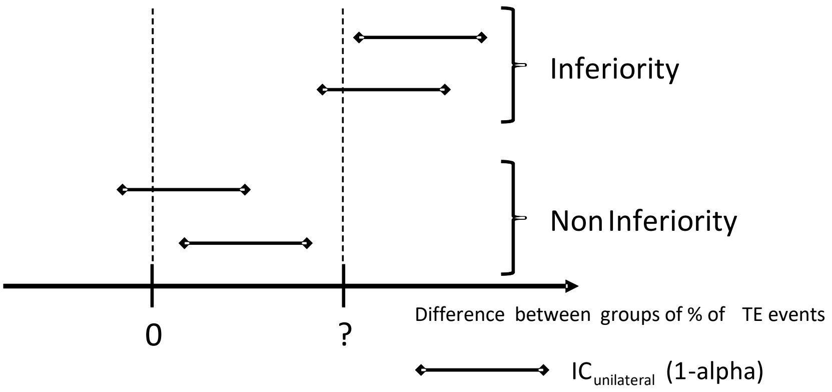

Secondary evaluation criteria:

Secondary criteria will be compared between groups on the ITT population and under superiority hypothesis.

Proportion of irradiative imaging, of onset of anticoagulation regimen, of hospital admission following the ED visit, of all causes hospital readmission at 3 month and of deaths from all causes at 3 months will be compared between groups by using a

generalized linear regression mixed models with Poisson distribution (log link). A random effect for each cluster will be considered and considered fixed effects will be period and strategy-by-period interaction If the number of events is sufficient, generalized linear regression mixed model with Bernoulli distribution (logit link) will be performed. Proportion difference between groups and its 95% confidence interval will be calculated.

The ED length of stay will be compared between the two periods by using a linear regression mixed model A random effect for each cluster will be considered and considered fixed effects will be period and strategy-by-period interaction. In case of non-normality distribution of the interest variable, a transformation could be realized.

Difference between groups of proportion of patients with diagnosed pulmonary embolism at 3 month follow-up excluding the isolated sub-segmental pulmonary embolism, among patients in whom PE has been initially ruled out, will be calculated as well its 95% confidence interval. If possible, generalized linear regression mixed models with Poisson distribution (log link) will be performed. A random effect for each cluster will be considered and considered fixed effects will be: period and strategy-by-period interaction If the number of events is sufficient, generalized linear regression mixed model with Bernoulli distribution (logit link) will be performed.

Proportion of patients with diagnosed thrombo-embolic event at 3 month follow-up (either a PE or a deep venous thrombosis), among patients in whom PE has been initially ruled out by the PEPS score will be described with its 95% confidence interval.

Sensitivity analysis will be performed on the per protocol population.

1.2 Calculation hypotheses for the number of subjects required and the result

In the recent recommendations from International Society of Thrombosis and Hemostasis on studies for PE diagnosis in the ED, it has been suggested that the **maximal acceptable failure rate of a tested strategy should not exceed 1.85% (Dronkers JTH 2017).**[29]

The recent large European prospective cohort studies on PE diagnosis (PROPER, PERCEPIC, YEARS, ADJUST-PE) reported a failure rate of 0.1-0.5%.

Sample size under non inferiority hypothesis:

With an anticipated failure rate of **0.5% in the control group, a non-inferiority margin set at 1.35%** (according to the ISTH recommendations so the upper bound of the 95% CI of the failure rate in the intervention group will not exceed 1.85%)beta=20% and one-sided alpha=2.5%, N1=857 patients are needed (East 6, Cytel).

Cluster design effect hypothesis:

- 20 clusters and 2 periods.
- Intraclass correlation coefficient (CCIC) = 0.018
- Inter period correlation (η) = 0.0115
- Mean cluster size for one period (m) = 22 patients
- Cluster design effect: D= (1+(m-1)xCCIC) - η = 1.37.

Sample size needed = D x N1 = 1174 patients.

Accounting for up to 5% of non-evaluable patients, we need to recruit 1234 patients.

Beside the non-inferiority analysis, it is of utmost importance that the upper bound of the 95%CI of the failure rate of the tested modified diagnostic strategy is below 1.85%, whatever the rate of the control group is.
Our retrospective study reported an anticipated failure rate at 0.85% but focused only on low risk patients. Therefore, **with an anticipated failure rate of 1% in the intervention period, the sample size of this group should be of at least 700 to respect the maximal upper bound of 1.85%.**
Therefore, for a **conservative** approach of these 2 conditions (non-inferiority margin at 1.35% and maximal upper bound of the 95%CI below 1.85%), **1400 (700 in each group) subjects will be needed in this study..**

1.3 Anticipated level of statistical significance

All tests will be performed at 5%.

1.4 Statistical criteria for termination of the study

Not applicable.

1.5 Method for taking into account missing, unused or invalid data

Missing value for the principal criteria will be considered as failure, whatever the period considered. Others missing data will not be replaced.

1.6 Management of modifications made to the analysis plan for the initial strategy

Modification made in analysis will be documented in the final report.

1.7 Choice of individuals to include in the analyses

ITT population: all included patients according to the period assigned by the randomization to the center, regardless of the strategy effectively received by the patient.

Per protocol population: all included patients without major protocol deviation that would be:
- No respect of selection criteria,
- No respect of strategy assigned by randomization (cross-over for example),
- Missing value for the principal criteria,
- Other major protocol deviation identified during data review and freezing of data base.

1.8 Medico-economic evaluation

This first economic evaluation of the innovative diagnostic strategy of PE follows the recommendations from the French national health authority and the CHEERS statement for single-trial based studies
(http://www.has-sante.fr/portail/upload/docs/application/pdf/201111/guide_methodo_vf.pdf

79. Husereau D, Drummond M, Petrou S, et al. Consolidated Health Economic Evaluation Reporting Standards (CHEERS) statement. BMJ 2013;346:f1049.).

The economic evaluation has to consider 3 possibilities: cost minimization which would assume the equivalence of strategies on the effectiveness endpoint; incremental cost effectiveness /decremental cost effectiveness; or dominance. Given the non-inferiority hypothesis the primary analysis is a cost-minimization analysis prolonged by a cost-effectiveness sensitivity analysis on the cost per major event (death and TE event averted). (Briggs AH, O'Brien BJ. The death of cost-minimization analysis? Health Econ. 2001;10:179-

84.). The perspective of the analysis is the healthcare system and the time horizon 3 months. The cost minimization/cost effectiveness analysis will use the difference in the safety of each strategy as the effectiveness criterion in accordance with previous studies (Sterne JA, Bodalia PN, Bryden PA, Davies PA, López-López JA, Okoli GN, Thom HH, Caldwell DM, Dias S, Eaton D, Higgins JP, Hollingworth W, Salisbury C, Savović J, Sofat R, Stephens-Boal A, Welton NJ, Hingorani AD.Oral anticoagulants for primary prevention, treatment and secondary prevention of venous thromboembolic disease, and for prevention of stroke in atrial fibrillation: systematic review, network meta-analysis and cost-effectiveness analysis.Health Technol Assess. 2017 Mar;21(9):1-386).

The economic evaluation is based on the entire population of patients included in the trial.

Estimating resources and costs

This is a single study-based economic evaluation: resource utilization will be collected prospectively at the patient level using the study case report form supplemented by data from the hospital claims database for the entire duration of the study period.

Medical care costs for the index admission and in-trial follow-up period are assessed using a combination of resource-based and event-based methods. In-hospital resource utilization will be described based on diagnosis and procedural codes and length of stay for the entire duration of the index admission and subsequent hospital stays. The total length of stay and discharge information (DRG) will be extracted from the hospital information system for the index admission and event-related subsequent admissions during the 1 month follow up period. Length of stay in the ICU, step down units and conventional wards will be retrieved. In France, hospital costs will be assigned based on the Severity-Diagnosis Related Groups. Specific ICU costs are assigned based upon the total duration of ICU stay. The cost of each admission will be estimated from the national cost study, using the actual length of stay and the per diem cost stratified by DRG.
We will use the latest mean cost available (or tariff if costs are not available).

In Spain, some hospitals experiment DRGs and produce DRG-specific costs. These costs cannot be extrapolated at the national level. Other hospitals calculate cost per department (eg cardiology) per day, aggregated at the regional level for budgeting purposes. We will record length of stay for all cardiac-related admissions and DRGs in hospitals where the information is available and derive average daily hospital costs (regional) to be used in cost comparisons.

Out of hospital resources will be estimated from the CRF and patients interviews during the follow up visits (35 days and 90 days). We will collect information on consultations, medications, laboratory tests. Out of hospital resources will be valued using the latest price/ tariff schedule. Missing cost data: it is expected that all in-hospital resources will be collected given the fact that a hospital admission is a serious adverse event.
Total costs under current diagnostic and treatment protocols will be estimated for each patient for the index attendance/admission and over 3 months of follow-up.

Discount rate

In view of the short duration of this study, costs and benefits are not discounted.

Cost effectiveness study

The primary outcome measure for the economic evaluation is 3-month cumulated adverse events defined as death or TE.

Data analysis

The unit of analysis is the patient. Costs will be presented as means with 2.5 to 97.5% bootstrapped intervals. Between-group comparisons of costs will be performed using the bootstrap t-test. A joint comparison of costs and effects will be performed by nonparametric bootstrapping with 1,000 resamples and the probabilities of better/worse performance of modified diagnostic algorithm and higher/lower costs will be determined.
